# Global burden of the COVID-19 associated patient-related delay in emergency healthcare: a panel of systematic review and meta-analyses

**DOI:** 10.1186/s12992-022-00836-2

**Published:** 2022-06-08

**Authors:** Vahid Mogharab, Mahshid Ostovar, Jakub Ruszkowski, Syed Zohaib Maroof Hussain, Rajeev Shrestha, Uzair Yaqoob, Poorya Aryanpoor, Amir Mohammad Nikkhoo, Parasta Heidari, Athar Rasekh Jahromi, Esmaeil Rayatdoost, Anwar Ali, Farshid Javdani, Roohie Farzaneh, Aref Ghanaatpisheh, Seyed Reza Habibzadeh, Mahdi Foroughian, Sayyed Reza Ahmadi, Reza Akhavan, Bita Abbasi, Behzad Shahi, Arman Hakemi, Ehsan Bolvardi, Farhad Bagherian, Mahsa Motamed, Sina Taherzadeh Boroujeni, Sheida Jamalnia, Amir Mangouri, Maryam Paydar, Neda Mehrasa, Dorna Shirali, Francesco Sanmarchi, Ayesha Saeed, Narges Azari Jafari, Ali Babou, Navid Kalani, Naser Hatami

**Affiliations:** 1 Department of Pediatrics, Jahrom University of Medical Sciences, Jahrom, Iran; 2 Research Center for Non-Communicable Diseases, Jahrom University of Medical Sciences, Jahrom, Iran; 3 Department of Nephrology, Transplantology and Internal Medicine, Faculty of Medicine, Medical University of Gdańsk, Gdańsk, Poland; 4 Department of Pathophysiology, Faculty of Medicine, Medical University of Gdańsk, Gdańsk, Poland; 5 ST1/Clinical Teaching Fellow, Norfolk and Norwich University Hospital, Norwich, United Kingdom; 6 Palliative Care and Chronic Disease Unit, Green Pasteur Hospital, Pokhara, Nepal; 7 Postgraduate trainee, Surgical Department, Hamdard University Hospital Karachi, Karachi, Pakistan; 8 Department of Epidemiology and Health Statistics, Xiangya School of Public Health, Central South University, Changsha, China; 9 Hunan Provincial Key Laboratory of Clinical Epidemiology, Xiangya School of Public Health, Central South University, Changsha, China; 10 Department of Emergency Medicine, Faculty of Medicine, Mashhad University of Medical Sciences, Mashhad, Iran; 11 Department of Radiology, Faculty of Medicine, Mashhad University of Medical sciences, Mashhad, Iran; 12 Department of Emergency Medicine, Faculty of Medicine, Zahedan University of Medical Sciences, Zahedan, Iran; 13 Department of Emergency Medicine, Babol University of Medical Sciences, Babol, Iran; 14 Department of Psychiatry, Tehran University of Medical Sciences, Tehran, Iran; 15 Medical Journalism Department, Shiraz University of Medical Sciences, Shiraz, Iran; 16 Fellowship of Vascular Surgery and Endovascular Therapy, Division of Vascular Surgery and Endovascular Therapy, Department of General Surgery, Sina Hospital, Tehran University of Medical Sciences, Tehran, Iran; 17 Shiraz Azad University, Dental Branch, Shiraz, Iran; 18 Department of Biomedical and Neuromotor Sciences, Alma Mater Studiorum, University of Bologna, Bologna, Italy; 19 Department of Biochemistry, Government College University, Faisalabad, Pakistan; 20 Neuroscience Research Department Center, Kashan University of Medical Science, Kashan, Iran; 21 Pharmaceutical Sciences Department, College of Pharmacy, Gulf Medical University, Ajman, United Arab Emirates

**Keywords:** COVID-19, SARS-COV-2, Pandemic, Emergency department

## Abstract

**Background:**

Apart from infecting a large number of people around the world and causing the death of many people, the COVID-19 pandemic seems to have changed the healthcare processes of other diseases by changing the allocation of health resources and changing people’s access or intention to healthcare systems.

**Objective:**

To compare the incidence of endpoints marking delayed healthcare seeking in medical emergencies, before and during the pandemic.

**Methods:**

Based on a PICO model, medical emergency conditions that need timely intervention was selected to be evaluated as separate panels. In a systematic literature review, PubMed was quarried for each panel for studies comparing the incidence of various medical emergencies before and during the COVID-19 pandemic. Markers of failure/disruption of treatment due to delayed referral were included in the meta-analysis for each panel.

**Result:**

There was a statistically significant increased pooled median time of symptom onset to admission of the acute coronary syndrome (ACS) patients; an increased rate of vasospasm of aneurismal subarachnoid hemorrhage; and perforation rate in acute appendicitis; diabetic ketoacidosis presentation rate among Type 1 Diabetes Mellitus patients; and rate of orchiectomy among testicular torsion patients in comparison of pre-COVID-19 with COVID-19 cohorts; while there were no significant changes in the event rate of ruptured ectopic pregnancy and median time of symptom onset to admission in the cerebrovascular accident (CVA) patients.

**Conclusions:**

COVID-19 has largely disrupted the referral of patients for emergency medical care and patient-related delayed care should be addressed as a major health threat.

**Supplementary Information:**

The online version contains supplementary material available at 10.1186/s12992-022-00836-2.

## Introduction

Coronavirus disease 2019 (COVID-19), the highly contagious infectious disease caused by severe acute respiratory syndrome coronavirus 2 (SARS-CoV-2) [1] was first reported on December 31, 2019, in Wuhan, China. One month later, on January 30, 2020, it was declared a global health emergency [2] compelling the World Health Organization (WHO) to declare it as a global pandemic on March 11, 2020. Globally, more than 6 million deaths are reported worldwide across 222 countries [3]. The virus affects the respiratory system and produces mild to severe respiratory illness, and might contribute to hospitalization, mechanical ventilation in intensive care units, and even death in some cases [4]. The severity of illness might get increased in people of older age, immunocompromised individuals, and those having pre-medical co-morbidities such as diabetes, cardiovascular disease, respiratory disease, and cancers [4, 5]. Since the world health organization declared COVID-19 a global pandemic, COVID-19 was not just a health threat but its prolonged national lockdowns and modified lifestyle of people have affected various aspects of almost every sector’s life. For example, it reduced students’ access to education, increased food insecurity to millions of people, increased poverty, worsened mental health of both the healthcare professionals and the general population, and increased the burden on healthcare services [3, 6].

Healthcare services utilization at the inpatient, outpatient, and emergency departments settings dropped due to the restrictive measures [7, 8]. Moreover, plenty of literature reported a reduction in the emergency department (ED) visits during the pandemic period [9, 10]. Diagnostic delays caused by the COVID-19 are mentioned to cause a major rise in the incidence of preventable cancer deaths in England [11]. Another report has approximated that 41% of individuals in the United States have postponed or avoided medical care, including urgent (12%) or non-urgent care (32%) [12]. Emergency medical care or urgent care, being provided by ED for individuals who arrive at the hospital, is defined as “Acute illness or damage that threatens life or function and needs prompt medical intervention. The patient would get hurt if there would be a delay” [13]. ED is responsible for stabilizing patients with life-threatening conditions and arrangement of admission of patients to special care facilities [13]. Healthcare avoidance is a type of patient disengagement that leads them to delay seeking medical care [14]. In some circumstances in the COVID-19 era, people experiencing urgent medical emergencies had been avoiding healthcare services due to the fear of contagion. Additionally, the EDs have also seemed to give lesser priority to non-COVID-19 patients comparatively [15]; while emergency medical health services are equally important irrespective of suffering from COVID or not. This reduction in the overall healthcare services utilization might worsen health outcomes for patients with other chronic diseases or acute medical emergencies [16]. Some studies also reported delayed emergency medical care in the case of pre-hospital services like the response to out-of-hospital cardiac arrest [17]. Others showed that the untimely and improper management of emergency medical needs increased morbidity and mortality of non-COVID-19 patients during the pandemic [11, 12, 15, 16]. These dysfunctions in healthcare management may delay the achievement of the Sustainable Development Goals (SDG) published by the United Nations. Indicators of sustainable development seek to ensure long-term stability in the economy, health, education, and the environment [18]; while it seems that COVID-19 have been imposing burdens of health financing on other aspects of SDG and even influencing significant portions of the healthcare system itself, in non-COVID-19 diseases care. As recently many studies have paid attention to the impacts of the pandemic on non-COVID-19 diseases management, reviewing these studies is needed for developing policies for shaping the normal post-pandemic healthcare system. As a response, we should immediately identify factors linked to healthcare delays, especially in urgent care, that are related to higher mortality and morbidity rates. These factors might be related to the healthcare system as well as pre-hospital services or long wait times in the emergency department or might be due to patient-related factors as well as avoidance of care due to fear of COVID-19. Therefore, the aim of this study is to evaluate the impact of the COVID-19 pandemic on medical emergencies and time-sensitive emergency health conditions that require urgent care within a specified time to avoid mortality and morbidity. This study will help to understand, identify and document the impacts of the global COVID-19 pandemic on the emergency healthcare services, and provide valuable evidence to improve policy and management of emergency medical care in the context of a global pandemic.

## Methods

### Study question

This study aims to evaluate the COVID-19 pandemic impact on the time-sensitive emergency health condition. The PICO (Population, Intervention, Comparison, and Outcomes) conceptualized for this study is shown in Table 1. The population of interest is healthy/stable patients being visited in ED for an emergency condition. The ED is responsible for stabilizing patients’ vital signs and providing the necessary medical consultations for patients to enter special wards or operating rooms. Particularly, ED physicians make consultations with specialties in General Medicine (Neurology, Cardiology, Nephrology, Gastrointestinal, Endocrinology, Rheumatology), General Surgery, Pediatrics, Obstetrics, gynecology, and Urology. We considered these classifications to comprehensively include all possible emergency conditions. We limited the analysis to conditions with a specific golden time/hour or any outcome showing the incidence of delayed care (for example orchiectomy is preventable for testicular torsion if being treated at golden hours). The phrase “golden hour” was invented to emphasize the importance of timely emergency care in a time window that treatment would most prevent mortality and morbidity [28]. Outcomes of interest were the prevalence of failure/disruption of treatment due to delayed referral and onset to hospital door time, and onset to treatment time. We compared two time periods, before and during COVID-19.

**Table 1:** PICO method for study questions

PICO Evidence-based study concepts						Reference
P: Population of interest	Emergencies in different ED consultations which needs a timely intervention	Neurology	Meningitis	Acute ischemic stroke	Seizures	[19]
		Cardiology	Acute MI/ Acute Coronary Ischemia	Aneurysm	Aortic Dissection	[20]
			Cardiac Tamponade			
		Nephrology	polyangiitis and Wegener’s granulomatosis	Nephrotic syndrome		[21]
		Gastroenterology	Upper GI bleeding	Lower GI bleeding		[22]
		Endocrinology	Diabetic ketoacidosis (DKA)	Hypoglycemia	Acute adrenocortical insufficiency	[23]
			Phaeochromocytoma crisis	Acute Hypercalcaemia	Thyroid storm	
			Myxoedema coma	Acute pituitary apoplexy		
		Rheumatology	Polyarteritis nodosa	polyarteritis nodosa	Scleroderma	[21]
			polyangiitis and Wegener’s granulomatosis	Catastrophic antiphospholipid syndrome		
		General surgery	Acute abdominal conditions, including:		Respiratory obstruction, foreign bodies	[24]
			Incarcerated and Strangulated Inguinal Hernias	Bleeding from esophageal varices		
			Appendicitis	Pelvic infections with abscesses		
			Intestinal obstruction	Perforated typhoid ulcers	Surgical infections	
			Complications of peptic ulcer	Amebic liver abscess		
			Gall bladder and bile duct disease			
		Obstetrics & Gynecology	torsion of ovary	Ectopic Pregnancy		[25]
			pre-eclampsia and eclampsia	placenta praevia/placental abruption	Miscarriage	
			premature rupture of membranes			
		Urology	Acute Scrotum (torsion of testis)	Acute Urinary Retention	Severe Hematuria	[26]
			Lithiasis	Fournier Gangrene		
		Psychiatry	Suicide	Agitated and violent patients		[27]
I: Intervention	Disease specific intervention in golden time					
C: Control	Pre-COVID-19 outcomes in same centers per study					
O: Outcome	Prevalence of Failure / Disruption of treatment, Prevalence of disease complications due to delayed care, Onset to hospital door time, Onset to treatment time,					

Based on this concept, and using the National Confidential Enquiry into Patient Outcome and Death (NCEPOD) classification of intervention [29], diseases that need interventions that a reservation is being made before a routine hospitalization (elective intervention) and diseases that do not pose a threat to life, limb, or organ survival within a few days after deciding to conduct the intervention (expedited intervention) were not included in our study scope; whereas diseases that needed intervention immediately or within hours of the decision to operate were included in our study. But in-hospital timings like patient waiting time and delayed decision makings were waived in this study as our primary literature review did not show the feasibility of meta-analysis due to low data availability.

So, our study question was conceptualized to be “has the incidence of [endpoint marking delayed healthcare seeking] in [a medical emergency] been changed in comparison of patients referring to EDs before and during the COVID-19?” or “has the time of disease symptom onset to ED room been changed in comparison of patients referring to EDs before and during the COVID-19?”

This Systematic review study was performed based on the Preferred Reporting Items for Systematic Reviews and Meta-Analyses (PRISMA) guidelines. Selected populations of interest (the emergency condition) attributed MeSH terms were considered as main keywords. Search strings used for the selected conditions are listed in supplementary Table 1. In each panel, 2 independent researchers performed the literature review.

**Fig. 1:**
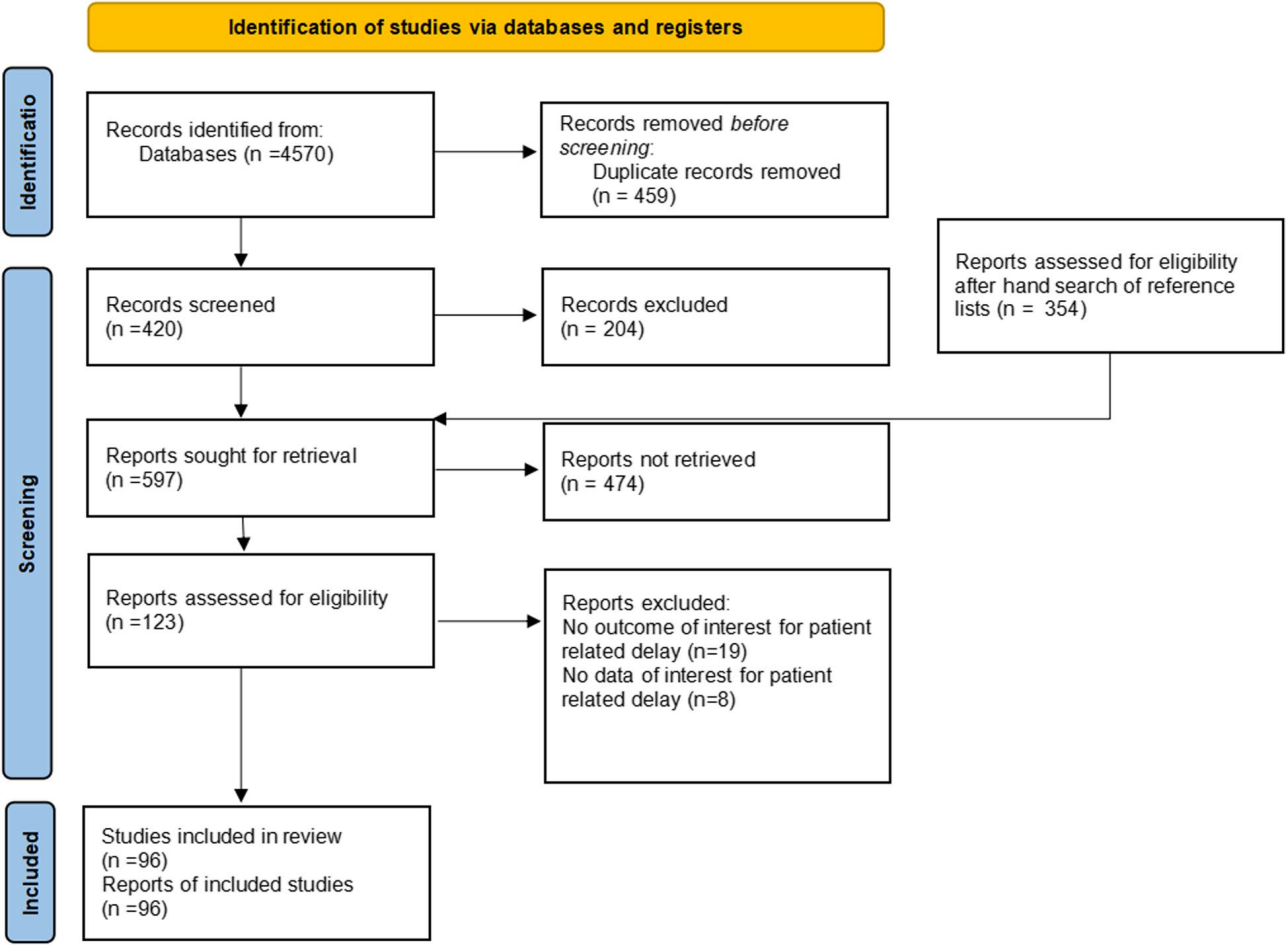
Prisma Flow chart of study the National Institutes of Health (NIH) Quality Assessment Tool (Quality Assessment Tool for Observational Cohort and Cross-Sectional Studies) was used to assess the quality of included studies and ranking studies in three categories of “good”, “fair”, and “poor”. (https://www.nhlbi.nih.gov/health-topics/study-quality-assessment-tools)

The inclusion criteria for studies in this study were english articles that had reported variables of interest before and during the COVID-19 pandemic in the same medical centers. After removing the duplicated search results, potentially relevant studies were collected for eligibility assessment. A third researcher judged the study in which the last two independent researchers didn’t agree to include. The search process is summarized in Fig. 1. Reference lists of studies were also hand queried for relevant references.

### Data extraction

In the case of the ACS panel, patient-related delay indicators were chosen to be the median time of symptom onset to first medical contact and symptom onset to administration in all ACS cases (STEMI and NSTEMI), and rate of delayed administration in STEMI cases (> 12 h). In acute appendicitis panel, perforated appendicitis rate, diagnosed in operation and later than 72 hours ED visit were considered as outcomes. In aneurismal SAH, vasospasm findings on CT angiography and The World Federation of Neurological Surgeons (WFNS) score higher than 3 and Fisher grade of higher than 2 (which is showing the amount of hemorrhage) were considered. Tissue plasminogen activator (rt-PA) administration rate and symptoms onset to ED door time was considered for stroke. Rupture of ectopic pregnancy, orchiectomy, and DKA presentation was chosen as indicators of delayed presentation in ectopic pregnancy, testicular torsion, and newly diagnosed T1DM panels, respectively. Study id, time frames, and country were also extracted.

### Analysis

Data of Studies with Quantitative outcomes of interest (time from onset to hospital or treatment) were collected and analyzed with Difference in Means or Difference of medians (DoM) in r packages. Data of studies with binary outcomes of interest (treatment failure or event of delayed care sought) were extracted in form of event rate in the total number of cases, before and during the COVID-19 pandemic. Binary data of rates were extracted as proportions of total study sample risk ratio was calculated to be pooled.

The Cochran Q test (two-test for heterogeneity) was used to assess the heterogeneity of the studies. I^2^ was used to calculate the percentage of total heterogeneity to total variability. A Q test with a *P* < 0.1 or an I^2^ statistic of greater than 60% was considered significant statistical heterogeneity. The random-effects model or fixed-effect model was used in case of heterogeneity presence or not, respectively. A 2-sided *P* < 0.05 was considered statistically significant. Publication Bias assessment was conducted by Funnel plot to depict publication bias. Egger’s bias test was used to determine asymmetry.

Relative change in disease incidence was visualized on a world map created using Datawrapper online tool (https://app.datawrapper.de) and it is based on data provided by studies reporting parallel timeframe of the pre-pandemic and pandemic period.

## Results

Following the literature review, 96 studies were included in the study in 7 panels for different medical conditions of (i) DKA rate in T1DM [8 studies]; (ii) Vasospasm rate in CT angiography [2 studies]; (iii) Orchiectomy rate in testicular torsion [6 studies]; (iv) rt-PA receiving rate in CVA patients [27 studies]; (v) Perforated appendicitis rate in acute appendicitis [20 studies]; (vi) rupture rate in ectopic pregnancy [8 studies]; and (vii) ACS patient-related delay [22 studies], as shown in Table 2. A total number of 139,542 patients were included in the before COVID-19 cohort and 84,601 in the COVID-19 cohort.

**Table 2:** Characteristics of included studies

	Study ID	ref	Before the COVID-19			During the COVID-19			Country	relative change^a^	Quality
			number of total cases	number of events	Time frame	number of total cases	number of events	Time frame			
DKA rate in T1DM	Atlas al.	[30]	204	86	2020	58	30	2017–2019	Australia	0.81%	good
	Ponmani al.	[31]	150	49	January and July, 2020	178	79	2019	UK	1.03%	good
	Rabbone al.	[32]	208	86	2020	160	61	20 February and 14 April 2019	Italia	0.51%	good
	Kamrath et al.	[33]	959	233	March 13 to May 13 2020	532	238	2019 and 2018	Germany	1.6%	good
	Bogale et al.	[34]	370	172	03/01/2020- and 09/14/2020	42	19	1/1/2017 to 2/28/2020	USA	0.51%	good
	Ho et al.	[35]	114	52	March 17 to August 31, 2020	107	73	2019	Canada	1.04%	good
	Gera al.	[36]	31	13	2020	33	21	1 March to 30 June, 2019	USA	1.1%	fair
	Lawrence al.	[37]	42	11	March to May,2020	11	8	2015–2019	Australia	2.51%	good
Vassospasm rate in CT angiography	Fiorindi et al.	[38]	179	14	March 9 to May 10, 2017–2018-2019	72	13	March 9 to May 10, 2020	Italy	2.23%	fair
	Aboukaïs et al.	[39]	28	21	March 1st, 2019 and April 26th, 2019	26	24	March 1st, 2019 and April 26th, 2020	France	0.48%	good
Orchiectomy rate in testicular torsion	Nelson et al.	[40]	77	13	1 January 2018–29 February 2020	17	5	1 March 2020–31 May 2020	USA	1.57%	good
	Littman et al.	[41]	47	21	2015 to 2019	20	5	March 15, 2020 to May 4, 2020	USA	0.11%	good
	Pogorelić et al.	[42]	68	11	January 1st, 2019 to March 10th, 2020	51	22	March 11th, 2020 to December 31st, 2020	Croatia	2.5%	good
	Holzman et al.	[43]	137	40	January 2019 through February 2020	84	34	March through July 2020	USA	1.09%	good
	Lee et al.	[44]	55	18	3/11/2018 to 10/1/2019	27	12	3/11/2020 to 10/1/2020	USA	1.03%	good
	Shields	[45]	79	30	March 1, 2015-December 31, 2019	38	19	March 1, 2020-December 31, 2020	USA	0.94%	good
rt-PA reciving rate in CVA patients	Xu et al.	[46]	153	53	December 1, 2019, and January 30, 2020	99	29	February 1, 2020, and March 31, 2020	China	0.5%	good
	Velilla-Alonso et al.	[47]	112	65	March 14 to May 14, 2019	83	36	March 14 to May 14, 2020	Spain	0.17%	good
	Aref et al.	[48]	118	17	whole study in December 7, 2019 and May 10, 2020; not clearly addresed	136	31	whole study in December 7, 2019 and May 10, 2020; not clearly addresed	Egypt	1.44%	fair
	Roushdy. et al.	[49]	151	16	February 15 to april 3, 2019	93	20	February 15 to april 3, 2021	Egypt	1.92%	good
	Katsanos et al.	[50]	8		March 17- april 30, 2019	12		March 17- april 30, 2020	Canada		fair
	Teo et al.	[51]	89	64	January 23, 2020–March 24, 2019	73	40	January 23, 2020–March 24, 2020	Hong Kong	0.04%	good
	Padmanabhan et al.	[52]	167	22	March 15th and April 14th, 2019	101	11	March 15th and April 14th, 2020	UK	0.69%	good
	D’Anna et al.	[53]	283	46	23rd March to 30th June 2019	235	27	23rd March to 30th June 2020	UK	0.54%	good
	Paliwal. et al.	[54]	206	25	from 1st November 2019 to 7th February 2020	144	24	from 7th February to 30th April 2020	Singapore	1.25%	good
	Tejada Meza et al.	[55]	492	178	March 9–May 3, 2020	304	97	December 30, 2019 - March 9, 2020	Spain	0.52%	good
	Agarwal et al.	[56]	634	195	6/1/2019–2/29/2020	120	38	03/012020–05/152020	US	0.72%	good
	Wallace et al.	[57]	2692	335	Jan 1–Feb 29, 2020	1225	149	Mar 20–Apr 25, 2020	US	0.85%	good
	Wu et al.	[58]	2354	1199	01/24/2019 to 04/29/2019	1281	791	01/24/2020 to 04/29/2020	china	0.7%	good
	Sevilis. et al.	[59]	15,226	1137	December 1, 2019, to March 15, 2020	11,105	88	March 15, 2020 to June 27, 2020	US	0.03%	good
	Tavanaei et al.	[60]	190	25	2019 (Mar 1 to Jun 1)	95	18	2020 (Mar 1 to Jun 1)	Iran	1.31%	good
	Srivastava et al.	[61]	39,113	4576	November 1, 2019 and February 3, 2020	41,971	4785	February 4, 2020 and June 29, 2020	US	0.86%	good
	Frisullo et al.	[62]	41	13	March–April 2019	52	7	March–April 2020	Italy	0.11%	fair
	Luo et al.	[63]	377	293	January 2019 to May 2019	315	231	January 2020 to May 2020	China	0.17%	good
	Bhatia et al.	[64]	1237	182	February and July 2019	1312	230	February and July 2020	India	1.04%	fair
	Cummings et al.	[65]	5239	656	March 2019 to February 2020	613	95	March to April 2020	US	1.11%	good
	Rinkel et al.	[66]	407	59	October 21st–December 8th 2019	309	50	March 16th–May 3th 2020	Netherlands	0.97%	good
	Ramos-Pachón et al.	[67]	1033	300	March 15–May 2, 2020	805	177	March 15–May 2, 2020	Spain	0.47%	good
	Meza et al.	[68]	225	52	30 December 2019 to 14 march, 2020	93	20	15 march, 2020 to 4 May 2020	Spain	0.7%	fair
	Velilla-Alonso et al.	[47]	112	65	March 14 to May 14, 2019	83	36	March 14 to May 14, 2020	Spain	0.17%	good
	Nagamine et al.	[69]	37	15	March 1–April 30, 2019	36	10	March 1–April 30, 2020	US	0.28%	good
	Siegler et al.	[70]	1491	124	March 1, 2019, and July 31, 2019	1464	54	March 1, 2020, and July 31, 2020	US	0.36%	good
	Wang et al.	[71]	320	20	12/1/19–03/11/20	255	30	03/12/20–06/30/20		1.82%	fair
Perforated appendicitis rate in acute appendicitis	Yang et al.	[72]	129	10	January to September 209	106	19	January to September 2020	china	0.25%	good
	Zhou et al.	[73]	121	10	2019	81	15	2020	china	0.26%	good
	Tankel et al.	[74]	237	31	31 December 2019–18 February 2020	141	29	19 February 2020–07 April 2020	Israel	0.43%	good
	Orthopoulos et al.	[75]	199	50	February 1–March 15, 2020/ 2019/ 2018	40	25	March 16, 2020–April 30, 2020	USA	−0.22%	good
	Kumaira Fonseca et al.	[76]	82	12	March and April 2019	36	11	March and April 2020	Brazil	0.17%	good
	Turanli et al.	[77]	145	31	March 1st,2019–February 29th, 2020	59	12	March 1st, 2020–May 31st, 2020	Turkey	0.85%	good
	Wang et al.	[78]	48	6	January 21, 2018 to May 6, 2018, and January 21, 2019 to May 6, 2019	32	10	January 2020 to May 2020	USA	0.09%	fair
	Jäntti et al.	[79]	127	22	1 February 2020 and 30 April 2020; first 6 weeks	99	31	1 February 2020 and 30 April 2020; second 7 weeks	Finland	0.24%	good
	Lisi et al.	[80]	34	9	February 2019 and December 2019	27	16	February 2020 and December 2020	Italy	−0.15%	good
	Burgard et al.	[81]	241	37	March 12 to June 6, 2017, 2018, and 2019	65	21	March 12 to June 6, 2020	switzerland	0.15%	good
	Antakia et al.	[82]	110	22	November 1, 2019 to March 10, 2020	59	12	March 10, 2020 to July 5, 2020	UK	0.78%	good
	Finkelstein et al.	[83]	59	10	March to May 2019	48	16	March to May 2020	USA	0.18%	good
	Sartori et al.	[84]	791	76	march-April 2019	546	87	arch-April 2020	Italy	0.44%	good
	Baral et al.	[85]	42	6	90 prior March 24 2020	50	10	90 days after March 242,020	Nepal	0.51%	fair
	Toale et al.	[86]	122	11	January 1st – March 25th	62	13	March 26th – May 31st	Ireland	0.22%	good
	Dreifuss et al.	[87]	65	11	April 1, 2020 and April 30, 2018, 2019	15	7	April 1, 2020 and April 30, 2020	Argentina	−0.1%	good
	Mor Aharoni et al.	[88]	60	5	1 March 2019 to 30 April 2019	74	0	April 1, 2020 and April 30, 2020	israel	NA	fair
	Neufeld et al.	[89]	840	181	December 1, 2019–March 10, 2020	91	25	March 11, 2020–May 16, 2020	usa	0.51%	good
	Scheijmans et al.	[90]	642	157	February and March 2019	607	179	February and March 2020	Netherlands	0.53%	good
	Somers et al.	[91]	69	4	12/03/2019 and 30/06/2019	40	8	12/03/2020 and 30/06/2020	Ireland	0.09%	good
rupture rate in ectopic pregnancy	Barg et al.	[92]	43	2	March 10–May 12, 2019	29	6	March 10–May 12, 2020	Israel	0.02%	good
	Dvash et al.	[93]	30	5	15 March and 15 June 2018, 2019	19	11	15 March and 15 June 2020	Israel	−0.29%	good
	Toma et al.	[94]	136	82	March 2019 and February 2020	62	50	March 2020 and June 2020	Delaware	−0.06%	fair
	Platts et al.	[95]	179	4	January 2019–June 2019	162	3	March 2020–August 2020	UK	1.19%	good
	Casadio et al.	[96]	201	52	January 1st 2014 - February 29th 2020	9	6	March 1st to 30th April 2020	Italy	−0.28%	fair
	Anteby et al.	[97]	208	23	February 27, 2020 to September 27, 2018, 2019	100	23	February 27, 2020 to September 27, 2020	Israel	0.25%	good
	Werner et al.	[98]	12	51	2019–2020	10	12	March 15th and May 17th, 2020	USA	2.34%	fair
	Dell’Utri et al.	[99]	9	20	February 24 th - May 31 th 2019	11	16	February 24 th - May 31 th 2020	Italy	0.07%	good
ACS patient-related delay	Tam et al.	[100]	48	–	February 1, 2018, to January 31, 2019	7	–	January 25, 2020, to February 10, 2020	China	NE	fair
	Fileti et al.	[101]	94	–	10 March and 10 April 2019,	72	–	10 March and 10 April 2020,	Italy	NE	good
	Mesnier et al.	[102]	664	–	Feb to Mar 16 2020	457	–	Mar 17 to Apri 122,020	France	NE	good
	Choudhary et al.	[103]	1488	–	(25 March to 24 April 2020	289	–	(25 January to 24 February 2020	India	NE	good
	Kwok et al.	[104]	33,255	–	1 January 2017 to 22 March 2020	683	–	23 March 2020 to 30 April 2020	UK	NE	good
	Erol et al.	[105]	1872	–	15-day registry (November 1–15, 2018	991	–	April 17–May 2, 2020	Turkey	NE	good
	Erol (b) et al.		1872	–		992	–			NE	
	Toner et al.	[106]	102	–	March 16–April 15 between 2014 and 2019	20	–	March 16–April 15, 2020	Australia	NE	good
	Li et al.	[107]	1092	–	2019 (Feb to Apr)	1038	–	2020 (Feb to Apr)	Taiwan	NE	fair
	Claeys et al.	[108]	260	–	March 13 to April 3 2019	188	–	March 13 to April 32,019	Belgium	NE	fair
	Trabattoni et al.	[109]	10	–	March 8 and April 10,2019	24	–	March 8 and April 10,2020	Italy	NE	fair
	Braiteh et al.	[110]	113	–	March/April of 2019	67	–	March/April 2020	USA	NE	good
	Cammalleri et al.	[110]	35	–	Mar 1 to Mar 31 2019	13	–	Mar 1 to Mar 31 2020	Italy	NE	good
	Scholz et al.	[111]	1329	–	Mar 2017–2019	387	–	Mar2020	Germany	NE	good
	Scholz (b) et al.		1330	–		388	–			NE	good
	Hauguel-Moreau et al.	[112]	63	–	2018–2019 from February 17 to April 26	16	–	March 17, 2020 in France (week 12)	France	NE	good
	Romaguera et al.	[113]	524	–	1 March to 19 April 2019	395	–	1 March to 19 April, 2020	Spain	NE	fair
	Romaguera (b) et al.		525	–		396	–			NE	
	Xiang et al.	[114]	626	–	4 weeks before January 24, 2020	236	–	4 weeks after January 24, 2020	China	NE	good
	Xiang (b) et al.		15,729	–	4 weeks before January 24, 2020	11,598	–	4 weeks after January 24, 2020	non-hubai	NE	good
	Yasuda et al.	[115]	274	–	January–July 2015–2019	64	–	January–July 2020	Japan	NE	good
	Sutherland et al.	[116]	145	–	1 March 2020 to 31 April 2020	108	–	1 March 2020 to 31 April 2020	Australia	NE	good
	Sutherland (b) et al.		145	–	1 March 2020 to 31 April 2021	82	–	1 July 2020 to 31 August 2020	Australia	NE	good
	Trabattoni et al.	[117]	386	–	Jan 1-Dec 31, 2019	599	–	Jan 1-Dec 31, 2020	Italy	NE	good
	Calvão et al.	[118]	80	–	March and April 2019	71	–	March and April 2020	Portugal	NE	good
	Chan et al.	[119]	908	–	23 March – 26 April 2015–2019	164	–	23 March – 26 April 2020	New Zealand	NE	good
	Nan et al.	[120]	158	–	between August 1, 2019, and January 22, 2020	52	–	January 23, 2020, and March 31, 2020	China	NE	good
	Tomasoni et al.	[121]	51	–	Jan 3 to Feb 20, 2020	34	–	Feb 21 to Apr 10, 2020	France	NE	good

^a^Relative change in event rate; *NE* not estimated

### Highlights of the results

We found significant changes in the pattern of patients’ referral to EDs in the case of ACS, aneurismal SAH, acute appendicitis, newly diagnosed T1DM, and testicular torsion with the emergence of the pandemic; while other medical emergencies did not show significant differences. Here the details of statistical analyses for pooling the studies are presented separately for each panel.

As shown in Table 2, 28 studies were eligible in the stroke panel; of which 21 studies were included in the time metrics meta-analysis of Differences of Medians (DoM) of symptoms onset to ED door, and 25 were included in the meta-analysis of the proportion of rt-PA administration. Based on the random-effects model, there were no significant differences in median time from symptoms onset to ED door between pre-and during-COVID-19 cohorts in CVA subjects (DoM = 15.67 min, 95% CI:-22.84 to 54.18 min; *P* = 0.425, supplementary Fig. 1). However, we found high heterogeneity between studies (I^2^ = 98.31%) with no evidence of publication bias (Funnel Plot Asymmetry *P* = 0.969, supplementary Fig. 2). We did not recognize any source to evaluate as a meta-regression model to explain the high amount of heterogenicity.

In the case of the proportion of rt-PA administration among all CVA patients, based on the random-effects model, with a high value of heterogenicity (I^2^ = 97.56%), there were no differences in the event rate of receiving rt-PA in pre-COVID-19 and COVID-19 cohorts (RR = − 0.11, 95% CI:-0.33 to 0.11; *P* = 0.0914; supplementary Fig. 3). We did not observe evidence of publication bias (*P* = 0.541, supplementary Fig. 4).

Nine studies had reported ACS symptom onset to first medical contact of which 3 studies had subgroups in different time frames that finally 12 study/sub-group data was entered meta-analysis. Meta-analysis using a random-effects model (I^2^ = 99.52%) revealed no significant difference in DoM of symptom onset to first medical contact (minutes) in comparison of pre-COVID-19 cohorts with COVID-19 cohorts (DoM = 65.71 min, 95% CI:-11.55 to 142.98; *P* = 0.0955); while there was a high possibility of publication bias or small study effects due to asymmetry of the funnel plot (*P* = 0.0281), supplementary Fig. 5. The trim-filling method was not successful in eliminating bias and after using the trim-fill method publication bias was still present; more advanced statistical methods are needed in the case of DoM.

Seven studies had reported symptom onset to first medical contact of which 1 study had subgroups in different time frames that finally 8 study/sub-group data was entered meta-analysis. Meta-analysis with random-effects model (I^2^ = 61.21%; Q(df = 7) = 18.91, *P* = 0.0085) revealed significant increase in DoM of symptom onset to administration (minutes) in comparison of pre-COVID-19 cohorts with COVID-19 cohorts (DoM = 30.94 min, 95% CI:12.919 to 48.966; *P* = 0.0008); with no evidence for publication bias or small study effects (*P* = 0.0892).

In neurosurgery panel, aneurismal subarachnoid hemorrhage was chosen as emergency condition in which delayed health care sought was considered as vasospasm finding on CT angiography, Fisher grade > 2, and WFNS > 3. There were only 2 eligible studies. Due to I^2^ = 0.0% (Q(df = 1) = 0.0153, *P* = 0.901), we preferred to perform the meta-analysis. In a fixed effect model, there was a powerful statistically significant increased rate of vasospasm finding on CT angiography in comparison of Pre-COVID-19 and COVID-19 cohort (RR = 1.575, 95% CI:0.72 to 2.42; *P* = 0.003), as shown in supplementary Fig. 6; but findings were not statistically significant in case of Fisher grade > 2 (RR = -0.0064, 95% CI: − 0.2196 to 0.2068, *P* = 0.9533, I^2^ = 0.0%), as shown in supplementary Fig. 7; and WFNS > 3 (RR = 0.3088, 95% CI:-0.2631 0.8807, *P* = 0.2899, I^2^ = 42.40%, [Q(df = 1) = 1.7362, *P* = 0.1876]), shown in supplementary Fig. 8.

In the urology panel, in the case of testicular torsion, 6 studies were selected to be included in the meta-analysis of orchiectomy rate among testicular torsion cases, being age limited to pediatric cases to decrease the heterogeneity. In a fixed-effects model, with heterogeneity of 3%, RR was estimated to be 0.259 (95% CI:0.026 to 0.492; *P* = 0.029, supplementary Fig. 9) and no publication bias evidence (regression test for funnel plot asymmetry *p* = 0.883, supplementary Fig. 9). This was indicating a statistically significant rise in the rate of orchiectomy rate among testicular torsion in COVID-19 cohorts compared to pre-COVID-19.

In Endocrinology/pediatrics panel, in the case of newly diagnosed type 1 diabetes mellitus (T1DM), 8 studies were included in the meta-analysis of DKA presentation rate among T1DM cases, being age limited to pediatric cases to decrease the heterogeneity. Using a random-effects model, RR was estimated to be 0.224 (95% CI:0.062 to 0.38; *p* = 0.0065) and no publication bias evidence (regression test for funnel plot asymmetry *P* = 0.915, supplementary Fig. 10). The results presented in individual studies were moderately heterogeneous (I^2^ = 49.37%, Q(df = 7) = 14.98, *P* = 0.0362, supplementary Fig. 11). This shows a statistically significant increase in the rate of DKA presentation rate among T1DM patients, comparing pre-COVID-19 and COVID-19 cohorts.

In Obstetrics and gynecology panel, in the case of ectopic pregnancy, 8 studies were selected to be included in the meta-analysis of rupture of ectopic pregnancy rate among all ectopic pregnancy cases. In a random-effects model, with heterogeneity of 56.20% (Q(df = 7) = 17.0353, *P* = 0.0172, supplementary Fig. 12), RR was estimated to be 0.112 (95% CI:0.0248 to 0.201; *p* = 0.0065); but there was potential possibility of publication bias (regression test for funnel plot asymmetry *P* = 0.0121, supplementary Fig. 13). So, using the trim and fill method, 4 studies were filled, and the final RR was 0.0670 (CI95%: − 0.0064 to 0.1404; *p* = 0.0734, supplementary Figs. 14 and 15). So, there were no significant changes in the rate of EP rupture before and during the pandemic.

In the general surgery panel, in the case of acute appendicitis, 20 studies were selected to be included in the meta-analysis of Perforated appendicitis rate among all acute appendicitis cases, diagnosed based on post-operation findings. To minimize possible heterogeneity, adult-aged studies were included. In a Fixed- Effects Model, with heterogeneity of 18.59%, RR was estimated to be 0.362(CI95%:0.2549 to 0.4690; *p* < .0001; supplementary Fig. 16) and no publication bias evidence (regression test for funnel plot asymmetry *p*-value = 0.242; supplementary Fig. 17). This shows a statistically significant increase in the rate of the perforation rate among acute appendicitis patients, comparing pre-COVID-19 and COVID-19 cohorts. of 20 selected articles, 3 studies reported late symptom onset to ED referral rate in case of later than 72 hours ED visit to symptom onset time. In a meta-analysis of later than 72 h referral, using a random-effects model, with a heterogeneity of 75.32%, RR was estimated to be 0.641(CI95%: − 0.6104 to 1.8938; *p* = 0.315, supplementary Figs. 18 and 19). There were no significant changes in the rate of late referral (Table 3).

**Table 3:** Meta-analysis results

Panel	Outcome of interest	n	I^**2**^	Estimate	P
CVA	symptoms onset to ED door time	21	98.31%	DoM = 15.67 min, 95% CI:-22.84 to 54.18	0.4252
	rt-PA administration	25	97.56%	RR = −0.11, 95% CI:-0.33 to 0.11	0.0914
ACS	symptom onset to first medical contact^a^	12	99.52%	DoM = 65.71 min, 95% CI:-11.55 to 142.98	0.0955
	symptom onset to administration	8	61.21%	DoM = 30.94 min, 95% CI:12.919 to 48.966	0.0008
aneurismal SAH	Vassospasm finding on CT angiography	2	0.0%	RR = 1.575, 95% CI:0.72 to 2.42	0.003
	Fisher grade > 2	2	0.0%	RR = -0.0064, 95% CI: −0.2196 to 0.2068	0.9533
	WFNS > 3	2	42.40%	RR = + 0.3088, 95% CI:-0.2631 0.8807	0.2899
Acute appendicitis	Perforated appendicitis	20	18.59%	RR = + 0.362, 95% CI:0.2549 to 0.4690	<.0001
	later than 72 hours ED visit	3	75.32%	RR = + 0.641, 95% CI:-0.6104 to 1.8938	0.315
ectopic pregnancy	Rupture of ectopic pregnancy	8	56.20%	RR = + 0.112, 95% CI:0.0248 to 0.201 trim and filled: RR = + 0.0670, 95% CI: −0.0064 to 0.1404	trim and filled: 0.0734
newly diagnosed T1DM	DKA presentation	8	49.37%	RR = + 0.224, 95% CI:0.062 to 0.38	0.0065
Testicular Torsion	Orchiectomy	6	3%	RR = + 0.259, 95% CI:0.026 to 0.492	0.029

^a^Publication bias exist

**Fig. 2:**
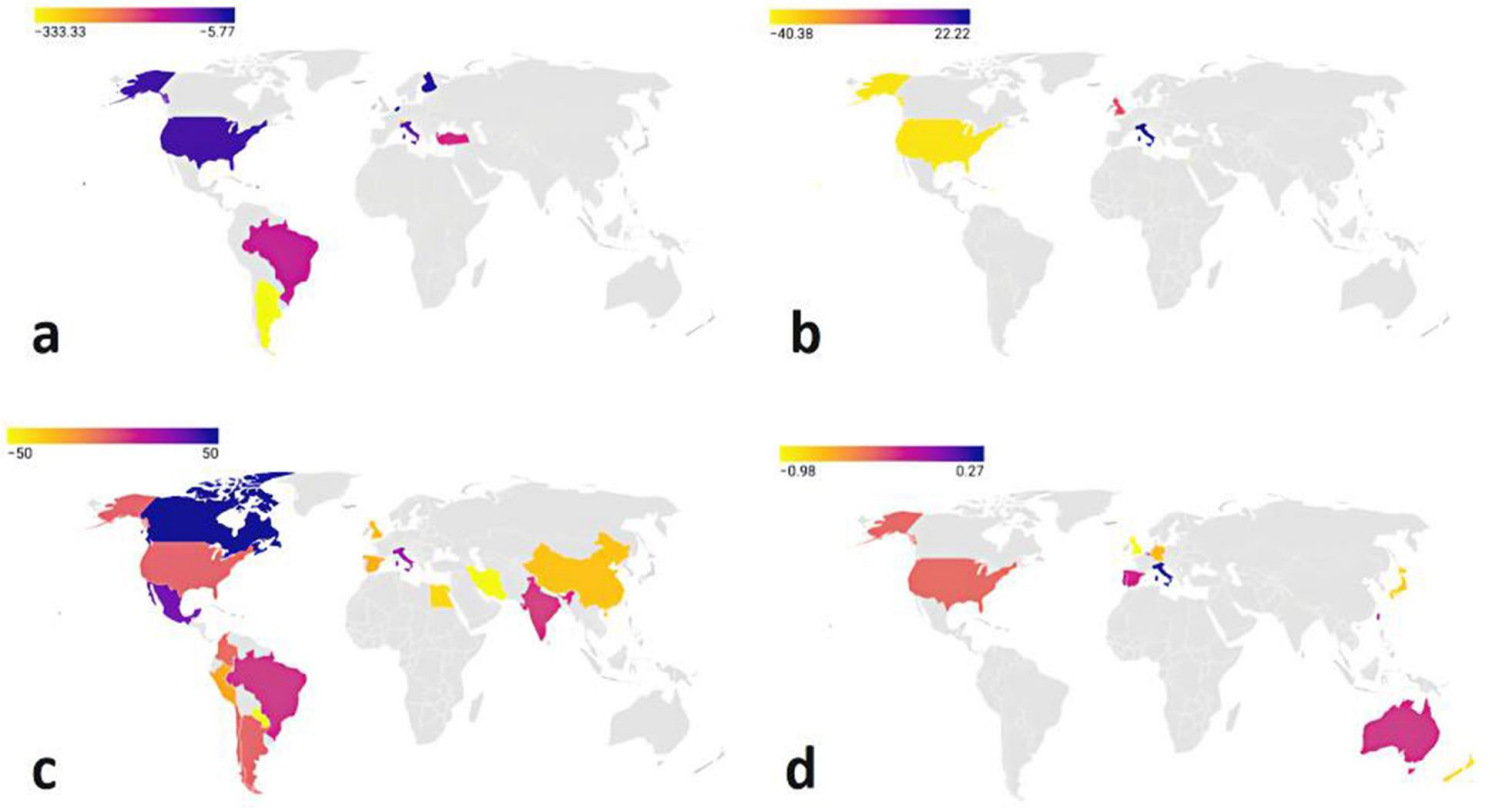
Schematic of the relative change of different diseases after the pandemic. Relative change of (**a**) acute appendicitis, (**b**) ectopic pregnancy, (**c**) CVA, and (**d**) ACS incidence during COVID-19 pandemic

Studies in which the time frames of pre-COVID-19 and COVID-19 cohorts were the same months of years were selected for estimation of the relative change of incidence. Based on the provided data which is shown in Table 2, the worldwide relative change of incidence was visualized in Fig. 2.

## Discussion

The sharp drop in emergency department admissions is mentioned in various studies [30–121]; however, according to our knowledge, no previous study has provided systematic evidence to support this view worldwide. We found that when comparing the pre-COVID-19 and COVID-19 cohorts of CVA patients, there were no substantial differences in the occurrence rate of obtaining rt-PA or the median time from symptom start to hospital room. In the case of ACS, the duration from symptom start to administration was significantly longer in pre-COVID-19 cohorts than in COVID-19 cohorts. When comparing the Pre-COVID-19 and COVID-19 cohorts of patients with aneurismal subarachnoid hemorrhage, there was a statistically significant higher prevalence of vasospasm on CT angiography; nevertheless, vasospasm indicates a delayed referral to hospital. In comparison to the pre-COVID-19 and COVID-19 cohorts, there was a statistically significant increase in the risk of perforation among acute appendicitis patients. There were no significant differences in the rate of ruptured Ectopic Pregnancy before and after the epidemic. When comparing the pre-COVID-19 and COVID-19 cohorts, there was a substantial rise in the rate of DKA presentation among T1DM patients as well as perforation rate among ectopic pregnancy patients. Similar to our study, Ojetti et al. attributed decreased admission of cardio-thoracic, gastroenterological, urological, otolaryngologic/ophthalmologic, and traumatological during the pandemic to fear of the virus, implying that patients with serious diseases did not seek treatment in the emergency department [122]. Toniolo et al. found that severe emergent cardiovascular diseases admissions were decreased during the pandemic in Italy [123]; a pooled analysis of similar studies showed a significant reduction in admission in a large comparison of 50,123 patients [124]. Several other studies are showing similar findings in many other medical conditions as well as surgical complaints [125], urological emergencies [126], and most other emergency department visits [127, 128].

All these studies unanimously warn of the danger of not paying attention to emergencies; while the decreased admission records could have happened due to various reasons. The changed use of the emergency department for the management of COVID-19 cases might be a reason that raises concerns about the disparities in healthcare. Previously, the concept of health disparities referred more to social differences and was addressing ethnicity and cultural minorities in the society, but COVID-19 era studies and the results of our study reveal a new concept of health disparities. Health disparities are one of the most important issues related to health policy and economics and are a major problem in the field of public health and social inequality. Health disparities are a general term used to denote the differences, variations, and disparities in access to health of individuals or groups [129]. While some researches show that elderly [130], Black populations, rural communities, and incarcerated populations [129] might experience inequality in healthcare; our previous study about Afghan refugees in Iran as a minor ethnicity [131] show that the need for active patient identification and treatment has lead widespread diagnostic and therapeutic measures of COVID-19 for patients with any social, economic, and cultural backgrounds and now we are facing a different side of the health disparity. Because the world’s healthcare market has been shifted to COVID-19 healthcare, governmental interventions are required to cover services for all people with other diseases, therefore, the study of inequality can provide accurate and reliable information on how health services are distributed to health planners and policymakers can determine the population groups that use the emergency services the least. In this study, we found some critical medical conditions that seem that the population affected by these diseases is receiving the required services lately; while statistics of mentioned studies might be showing patient-related decreased visits. In this study, we focused on patient-related delayed care-seeking. For this aim, known indicators of delayed healthcare sought were used to assess the hypothesis. Management of some emergency conditions is very time-critical and the best time to treat these diseases is called the Golden time or golden hour. We tried to address these medical conditions by pooling time metrics of patients’ referrals to emergency centers or in some cases, the final disease outcome that was showing delayed medical care were also compared before and during the pandemic. CVA and ACS were assessed mainly by time metrics. We found 25 studies that reported data of 7124 subjects experiencing CVA during the pandemic with more than seventy thousand subjects before the pandemic, time metrics of patient referral, and outcome of the rt-PA administration in proper time has not significantly changed; while as Fig. 2 shows ecological disparities exist. But, in the ACS panel, there was an increased symptom onset to administration time (30.94 min, 95% CI:12.919 to 48.966). We were aware of the possibility of the effect of the pre-hospital emergency care service delays and we also evaluated time to first medical contact that our analyses of time to first medical contact became worthless due to the possibility of bias and we were not able to address this by analytical methods.

Aneurysmal subarachnoid hemorrhage is a life-threatening condition that needs immediate medical attention. Delayed cerebral ischemia is a common issue that can lead to poor neurological results. The major cause of delayed cerebral ischemia is assumed to be cerebral vasospasm [132, 133]. We found that the presentation of SAH cases with vasospasm finding on CT angiography in comparison of Pre-COVID-19 and COVID-19 cohorts has shown a significantly higher incidence of vasospasm during the pandemic (OR = 1.575); while the number of studies included in the meta-analysis is low.

Our study revealed that DKA presentation in newly diagnosed T1DM patients has tended to get increase following the COVID-19 pandemic. It highlights the need for appropriate organization of healthcare resources, particularly for pediatric situations [134].

Due to parents’ concerns about the COVID-19 pandemic, visits to medical centers during the quarantine period may have occurred later than the pre-quarantine period [135]. Caregivers may mistakenly attribute symptoms to COVID-19 rather than DKA, resulting in an elevated severity of illness at the time of presentation with acute symptom start. Consequently, besides the organization of healthcare resources, the healthcare system has to educate patients and their families about life-threatening conditions and encourage them to look for help when needed.

Individuals will continue to experience fast metabolic decompensation, resulting in DKA, if the diagnosis of DM1 is delayed [136], as we saw during the COVID-19 pandemic. DKA is linked to increased morbidity and death, and our metanalysis suggests the necessity for focused public awareness efforts aimed at preventing DKA upon DM1 diagnosis by recognizing and treating symptoms early.

The lockdown has affected the availability of treatment services for patients with chronic diseases such as diabetes. Patients with diabetes have had a short- and long-term influence on glycemic parameters during catastrophes, according to previous studies, due to a lack of medical attention, proper meals, and prescriptions [137–139].

In other panels, we found a statistically significant higher perforation rate among acute appendicitis patients during the pandemic. No significant changes in the rate of ruptured ectopic pregnancy were seen before and after the pandemic. Also, rate of orchiectomy rate among testicular torsion was higher during the pandemic compared to before COVID-19. While Littman et al. [41] study did not find any delayed presentation of testicular torsion or its orchidectomy in comparison to pre-COVID-19 years; our study shows an increased pooled rate of orchidectomy testicular torsion during the COVID-19 pandemic emergence in the pooled analysis.

Many factors might justify this finding as well as the fear of COVID-19 infection and delayed referral to medical centers; There is a lot of unknown about Covid-19 disease for people; Therefore, these factors can be considered as an anxiety factor and have a negative effect on people psychologically. The psychological effects of the disease on ordinary people are such that the World Health Organization (WHO) has identified it as a risk factor for the mental health of society and has issued guidelines to prevent its destructive effects on the mental health of society [15, 140]. Various studies have shown that the prevalence of this disease and exposure to bad news published on social media about it, has increased anxiety and depressive symptoms, as well as impaired sleep quality [83]. One of the most vulnerable groups to bad news is children, and this bad news can increase their fear and anxiety, and such anxiety can affect their desire to go to the hospital. Since hospitals are at the forefront of the fight against this disease; They are one of the most infected places in terms of the presence of coronavirus and referring to it for the treatment of other diseases can be anxious for healthy people. Multiple pieces of research about pediatric acute appendicitis during the COVID-19 pandemic have clearly shown that staying at home due to public health safety instructions had a negative impact on those who had appendicitis. Several published studies found an increased risk of perforated appendicitis in pediatric patients during the COVID-19 pandemic compared with the pre-COVID-19 period [141]. Elective surgical procedures were discontinued in most centers during the COVID-19 outbreak. Surgical treatments were restricted to the treatment of patients who required immediate surgical or trauma attention. The attempts to reduce needless traffic through the healthcare institution resulted in a considerable decrease in emergency room patient visits. During the COVID-19 pandemic, the medical community noticed a marked increase in prolonged care for various medical emergencies, including pediatric surgical emergencies, which was documented in multiple papers.

### Limitations of the study

We only included PubMed as our searching database that some papers might not get included if being published in other indexing databases. While we attributed our study outcomes of interest to patient-related delayed healthcare, delay in performance of pre-hospital Emergency services and in-hospital long waiting times may have affected the study results. Also, delayed or wrong diagnosis and medical negligence might be the reason for delayed referral in some cases that are not discussed in the included papers.

## Conclusion

In addition to the dramatic changes that COVID-19 has posed to the trends of chronic diseases treatment and elective medical interventions, the treatment of some very urgent diseases has also been disrupted that is directly associated with unfortunate consequences such as death and disability. In this study, we tried to review the patterns of emergency medical care during the pandemic by focusing on the endpoints that are addressing delayed healthcare seeking. The reorganization of healthcare resources in response to the COVID-19 epidemic has resulted in inadvertent neglect of essential care, particularly in emergency medical circumstances. Following the COVID-19 pandemic, delayed care sought has tended to rise in some medical emergencies, according to our findings. Success in the early diagnosis of medical conditions that were addressed by our study (ACS, aneurismal SAH, acute appendicitis, newly diagnosed T1DM, and testicular torsion) depends to a large extent on people being aware of the early and warning signs of these diseases. It is necessary to comprehensively recall the community about the fundamentals of sickness symptoms, especially for acute diseases. Community education should raise the level of public awareness about the impact of acute medical conditions on health, as well as changes in the distribution of health resources during a pandemic or disaster. This should help them to be able to make decisions about their health even in certain circumstances. One sector involved in this is pre-hospital services and telemedicine that should properly guide people in choosing the best time and best medical center to refer to. Mass media can also influence people’s health behaviors and habits and the utilization of health services. Achieving all these ideals requires serious attention to health education in the structure of worldwide health sectors. Also, COVID-19 induced disparities in the allocation of health resources should be amended.

## Supplementary Information

**Additional file 1: Sup table 1.** Search strategy. **Supplementary Fig. 1.** Forrest plot of CVA symptoms onset to ED door time. **Supplementary Fig. 2.** Funnel plot of CVA symptoms onset to ED door time. **Supplementary Fig. 3.** Forrest plot of rt-PA administration proportion. **Supplementary Fig. 4**. Funnel plot of rt-PA administration proportion . **Supplementary Fig. 5**. Forest and Funnel plot of SAH Vasospasm (study 1, Fiorindi et al.; study 2, Aboukaïs et al.). **Supplementary Fig. 6.** Forest and Funnel plot of Fisher grade > 2 (study 1, Fiorindi et al.; study 2, Aboukaïs et al.). **Supplementary Fig. 7.** Forest and Funnel plot of WFNS > 3 (study 1, Fiorindi et al.; study 2, Aboukaïs et al.). **Supplementary Fig. 8.** Forrest plot of Orchiectomy rate in testicular torsion. **Supplementary Fig. 9.** Funnel plot of Orchiectomy rate in testicular torsion. **Supplementary Fig. 10.** Forrest plot of DKA presentation among newly diagnosed T1DM patients. **Supplementary Fig. 11.** Funnel plot of DKA presentation among newly diagnosed T1DM patients. **Supplementary Fig. 12.** Forrest plot of Perforated ectopic pregnancy. **Supplementary Fig. 13.** Funnel plot of Perforated ectopic pregnancy. **Supplementary Fig. 14.** trim-filled Forrest plot of Perforated ectopic pregnancy. **Supplementary Fig. 15.** trim-filled Funnel plot of Perforated ectopic pregnancy. **Supplementary Fig. 16.** Forest plot of perforated appendicitis proportion. **Supplementary Fig. 17.** Funnel plot of perforated appendicitis proportion. **Supplementary Fig. 18.** Forest plot of delayed appendicitis presentation. **Supplementary Fig. 19.** Funnel plot of delayed appendicitis presentation.

## Data Availability

There are no further data than presented in the manuscript and supplementary file.

## Abbreviations

ACS: Acute coronary syndrome
T1DM: Type 1 Diabetes Mellitus
CVA: Cerebrovascular accident
COVID-19: Coronavirus disease 2019
WHO: World Health Organization
ED: Emergency department
SDG: Sustainable Development Goals
DKA: Diabetic ketoacidosis
WFNS: World Federation of Neurological Surgeons
rt-PA: Tissue plasminogen activator
DoM: Differences of Medians
NE: Not estimated
NIH: National Institutes of Health
STEMI: ST-elevation myocardial infarction
EP: Ectopic pregnancy

## References

[1] World Health Organization. Coronavirus disease (COVID-19). [cited 2021 Nov 14]. Available from: https://www.who.int/health-topics/coronavirus#tab=tab_1 37184163

[2] World Health Organization. COVID-19 Public Health Emergency of International Concern (PHEIC) Global research and innovation forum. 2020 [cited 2021 Nov 14]. Available from: https://www.who.int/publications/m/item/covid-19-public-health-emergency-of-international-concern-(pheic)-global-research-and-innovation-forum

[3] Worldometer. Countries where Coronavirus has spread - Worldometer. [cited 2021 Nov 14]. Available from: https://www.worldometers.info/coronavirus/countries-where-coronavirus-has-spread/

[4] Ko JY, Danielson ML, Town M, Derado G, Greenlund KJ, Kirley PD (2021). Risk factors for coronavirus disease 2019 (COVID-19)–associated hospitalization: COVID-19–associated hospitalization surveillance network and behavioral risk factor surveillance system. Clin Infect Dis.

[5] Shahbazi F, Solgi M, Khazaei S. Predisposing risk factors for COVID-19 infection: a case-control study. Casp J Intern Med. 2020;11(Suppl 1):495. [cited 2021 Nov 14]. Available from: /pmc/articles/PMC7780876/. 10.22088/cjim.11.0.495 PMC7780876 33425266

[6] Onyeaka H, Anumudu CK, Al-Sharify ZT, Egele-Godswill E, Mbaegbu P (2021). COVID-19 pandemic: a review of the global lockdown and its far-reaching effects. Sci Prog.

[7] Xiao H, Dai X, Wagenaar BH, Liu F, Augusto O, Guo Y (2021). The impact of the COVID-19 pandemic on health services utilization in China: time-series analyses for 2016–2020. Lancet Reg Heal - West Pacific.

[8] Bodilsen J, Nielsen PB, Søgaard M, Dalager-Pedersen M, Speiser LOZ, Yndigegn T, et al. Hospital admission and mortality rates for non-covid diseases in Denmark during covid-19 pandemic: nationwide population based cohort study. BMJ. 2021;373 [cited 2021 Nov 16]. Available from: https://www.bmj.com/content/373/bmj.n1135. 10.1136/bmj.n1135 PMC8142604 34035000

[9] Jaehn P, Holmberg C, Uhlenbrock G, Pohl A, Finkenzeller T, Pawlik MT, et al. Differential trends of admissions in accident and emergency departments during the COVID-19 pandemic in Germany. [cited 2021 Nov 16]; Available from: 10.1186/s12873-021-00436-0. 10.1186/s12873-021-00436-0 PMC8022298 33823795

[10] Schwarz V, Mahfoud F, Lauder L, Reith W, Behnke S, Smola S (2020). Decline of emergency admissions for cardiovascular and cerebrovascular events after the outbreak of COVID-19. Clin Res Cardiol.

[11] Maringe C, Spicer J, Morris M, Purushotham A, Nolte E, Sullivan R (2020). The impact of the COVID-19 pandemic on cancer deaths due to delays in diagnosis in England, UK: a national, population-based, modelling study. Lancet Oncol.

[12] Czeisler MÉ, Marynak K, Clarke KE, Salah Z, Shakya I, Thierry JM (2020). Delay or avoidance of medical care because of COVID-19–related concerns—United States, June 2020. Morb Mortal Wkly Rep.

[13] Liu T, Sayre MR, Carleton SC (1999). Emergency medical care: types, trends, and factors related to nonurgent visits. Acad Emerg Med.

[14] Byrne SK (2008). Healthcare avoidance: a critical review. Holist Nurs Pract.

[15] Lazzerini M, Barbi E, Apicella A, Marchetti F, Cardinale F, Trobia G (2020). Delayed access or provision of care in Italy resulting from fear of COVID-19. Lancet Child Adolesc Heal.

[16] Santi L, Golinelli D, Tampieri A, Farina G, Greco M, Rosa S (2021). Non-COVID-19 patients in times of pandemic: emergency department visits, hospitalizations and cause-specific mortality in northern Italy. PLoS One.

[17] Baldi E, Sechi GM, Mare C, Canevari F, Brancaglione A, Primi R (2020). Out-of-hospital cardiac arrest during the Covid-19 outbreak in Italy. N Engl J Med.

[18] Odoch WD, Senkubuge F, Hongoro C (2021). How has sustainable development goals declaration influenced health financing reforms for universal health coverage at the country level? A scoping review of literature. Glob Health.

[19] McMullan JT, Knight WA, Clark JF, Beyette FR, Pancioli A (2010). Time-critical neurological emergencies: the unfulfilled role for point-of-care testing. Int J Emerg Med.

[20] Shahzad Anjum (2018). Systematic approach to acute cardiovascular emergencies, Essentials of Accident and Emergency Medicine, Ahmed Subhy Alsheikhly, IntechOpen, DOI: 10.5772/intechopen.74682. Available from: https://www.intechopen.com/chapters/60421.

[21] McGrath K, Slivka A (2003). Gastroenterologic emergencies: diagnosis and management. Gastroenterol Clin.

[22] Savage MW, Mah PM, Weetman AP, Newell-Price J (2004). Endocrine emergencies. Postgrad Med J.

[23] Kumar A, Marwaha V, Grover R (2003). Emergencies in rheumatology. J Indian Med Assoc.

[24] McCord C, Ozgediz D, Beard JH, Debas HT (2015). General surgical emergencies. Essent Surg: Dis Contr Priorities.

[25] Ramphal SR, Moodley J (2006). Emergency gynaecology. Best Pract Res Clin Obstetr Gynaecol.

[26] Reza MT, Autrán-Gómez AM, Tardío GU, Bolaños JA, Rivero JC (2020). Emergency surgery in urology during the COVID-19 pandemic. Int Braz J Urol.

[27] Sudarsanan S, Chaudhury S, Pawar AA, Salujha SK, Srivastava K (2004). Psychiatric emergencies. Med J Armed Forces India.

[28] Kotwal RS, Howard JT, Orman JA, Tarpey BW, Bailey JA, Champion HR (2016). The effect of a golden hour policy on the morbidity and mortality of combat casualties. JAMA Surg.

[29] National confidential enquiry into patient outcome and death. 2004, London: The NCEPOD classification of interventions [online], http://www.ncepod.org.uk/pdf/NCEPODClassification.pdf.

[30] Atlas G, Rodrigues F, Moshage MY, Welch J, White M, O'Connell MA. Presentation of paediatric type 1 diabetes in Melbourne, Australia during the initial stages of the COVID-19 pandemic. J Paediatr Child Health. 2020 Sep 22. 10.1111/jpc.15081. 10.1111/jpc.15081 PMC7537060 32959935

[31] Ponmani C, Sakka SD, Wickramarachchi CS, Ajzensztejn M, Kanumakala S, Redpath Y, et al. Characteristics of New-Onset Paediatric Type 1 Diabetes in the COVID-19 Pandemic–A Multicentre Perspective. Arch Dis Childh. 2021;106(Suppl 1):A28–9.

[32] Rabbone I, Schiaffini R, Cherubini V, Maffeis C, Scaramuzza A (2020). Has COVID-19 delayed the diagnosis and worsened the presentation of type 1 diabetes in children?. Diabetes Care.

[33] Kamrath C, Mönkemöller K, Biester T, Rohrer TR, Warncke K, Hammersen J (2020). Ketoacidosis in children and adolescents with newly diagnosed type 1 diabetes during the COVID-19 pandemic in Germany. Jama..

[34] Bogale KT, Urban V, Schaefer E, Bangalore KK. The impact of COVID-19 pandemic on prevalence of diabetic ketoacidosis at diagnosis of type 1 diabetes: a single-Centre study in Central Pennsylvania. Endocrinol Diabetes Metab. 2021;4(3):e00235. 10.1002/edm2.235 PMC7995137 34268453

[35] Ho J, Rosolowsky E, Pacaud D, Huang C, Lemay JA, Brockman N (2021). Diabetic ketoacidosis at type 1 diabetes diagnosis in children during the COVID-19 pandemic. Pediatr Diabetes.

[36] Gera S, Longendyke RL, Minich NM, Malay S, Wood JR. The COVID-19 pandemic and associated worsening of diabetic ketoacidosis presentation in youth. Diabet Med. 2021:e14610. 10.1111/dme.14610 PMC8236905 34053098

[37] Lawrence C, Seckold R, Smart C, King BR, Howley P, Feltrin R (2021). Increased paediatric presentations of severe diabetic ketoacidosis in an Australian tertiary Centre during the COVID-19 pandemic. Diabet Med.

[38] Fiorindi A, Vezzoli M, Doglietto F, Zanin L, Saraceno G, Agosti E, et al. Aneurismal subarachnoid hemorrhage during the COVID-19 outbreak in a hub and spoke system: observational multicenter cohort study in Lombardy, Italy. Acta Neurochirurgica. 164(1):141–50. 10.1007/s00701-021-05013-9 PMC8542653 34694465

[39] Aboukaïs R, Devalckeneer A, Boussemart P, Vromant A, Bricout N, Verdin MF (2021). Impact of COVID-19 pandemic on patients with intracranial aneurysm rupture. Clin Neurol Neurosurg.

[40] Nelson CP, Kurtz MP, Logvinenko T, Venna A, McNamara ER (2020). Timing and outcomes of testicular torsion during the COVID-19 crisis. J Pediatr Urol.

[41] Littman AR, Janssen KM, Tong L, Wu H, Wang MD, Blum E (2021). Did COVID-19 affect time to presentation in the setting of pediatric testicular torsion?. Pediatr Emerg Care.

[42] Pogorelić Z, Milanović K, Veršić AB, Pasini M, Divković D, Pavlović O, et al. Is there an increased incidence of orchiectomy in pediatric patients with acute testicular torsion during COVID-19 pandemic?-A retrospective multicenter study. J Pediatr Urol. 2021;17(4):479.e1-479.e6. 10.1016/j.jpurol.2021.04.017. 10.1016/j.jpurol.2021.04.017 PMC8087574 33994321

[43] Holzman SA, Ahn JJ, Baker Z, Chuang KW, Copp HL, Davidson J, et al. A multicenter study of acute testicular torsion in the time of COVID-19. J Pediatr Urol. 2021;17(4):478.e1-478.e6. 10.1016/j.jpurol.2021.03.013. 10.1016/j.jpurol.2021.03.013 PMC7977032 33832873

[44] Lee AS, Pohl HG, Rushton HG, Davis TD (2021). Impact of COVID-19 pandemic on the presentation, management and outcome of testicular torsion in the pediatric population-an analysis of a large pediatric center. Can J Urol.

[45] Shields LBE, Daniels MW, Peppas DS, White JT, Mohamed AZ, Canalichio K, et al. Surge in testicular torsion in pediatric patients during the COVID-19 pandemic. J Pediatr Surg. 2021;S0022-3468(21):00497-8. 10.1016/j.jpedsurg.2021.07.008. 10.1016/j.jpedsurg.2021.07.008 PMC9282895 34392971

[46] Gu S, Dai Z, Shen H, Bai Y, Zhang X, Liu X, et al. Delayed stroke treatment during COVID-19 pandemic in China. Cerebrovasc Dis. 2021. 10.1159/000517075. 10.1159/000517075 PMC8339026 34247153

[47] Velilla-Alonso G, García-Pastor A, Rodríguez-López Á, Gómez-Roldós A, Sánchez-Soblechero A, Amaya-Pascasio L (2021). Acute stroke care during the COVID-19 pandemic: reduction in the number of admissions of elderly patients and increase in Prehospital delays. Cerebrovasc Dis.

[48] Aref HM, Shokri H, Roushdy TM, Fathalla F, El Nahas NM (2021). Pre-hospital causes for delayed arrival in acute ischemic stroke before and during the COVID-19 pandemic: a study at two stroke centers in Egypt. PLoS One.

[49] Roushdy TM, El Nahas NM, Aref HM, Georgy SS, Zaki AS, Bedros RY (2020). Stroke in the time of coronavirus disease 2019: experience of two university stroke centers in Egypt. J Stroke.

[50] Katsanos AH, de Sa BD, Al-Qarni MA, Shawawrah M, McNicoll-Whiteman R, Gould L (2021). In-hospital delays for acute stroke treatment delivery during the COVID-19 pandemic. Can J Neurol Sci.

[51] Teo KC, Leung WC, Wong YK, Liu RK, Chan AH, Choi OM (2020). Delays in stroke onset to hospital arrival time during COVID-19. Stroke..

[52] Padmanabhan N, Natarajan I, Gunston R, Raseta M, Roffe C (2021). Impact of COVID-19 on stroke admissions, treatments, and outcomes at a comprehensive stroke Centre in the United Kingdom. Neurol Sci.

[53] D'Anna L, Brown M, Oishi S, Ellis N, Brown Z, Bentley P (2021). Impact of national lockdown on the hyperacute stroke care and rapid transient ischaemic attack outpatient service in a comprehensive tertiary stroke Centre during the COVID-19 pandemic. Front Neurol.

[54] Paliwal PR, Tan BY, Leow AS, Sibi S, Chor DW, Chin AX (2020). Impact of the COVID-19 pandemic on hyperacute stroke treatment: experience from a comprehensive stroke Centre in Singapore. J Thromb Thrombolysis.

[55] Tejada Meza H, Lambea Gil Á, Sancho Saldaña A, Martínez-Zabaleta M, Garmendia Lopetegui E, López-Cancio Martínez E (2020). Impact of COVID-19 outbreak in reperfusion therapies of acute ischaemic stroke in Northwest Spain. Eur J Neurol.

[56] Agarwal S, Scher E, Rossan-Raghunath N, Marolia D, Butnar M, Torres J (2020). Acute stroke care in a new York City comprehensive stroke center during the COVID-19 pandemic. J Stroke Cerebrovasc Dis.

[57] Wallace AN, Asif KS, Sahlein DH, Warach SJ, Malisch T, LaFranchise EF (2021). Patient characteristics and outcomes associated with decline in stroke volumes during the early COVID-19 pandemic. J Stroke Cerebrovasc Dis.

[58] Wu Y, Chen F, Wang Z, Feng W, Liu Y, Wang Y (2020). Reductions in hospital admissions and delays in acute stroke care during the pandemic of COVID-19. Front Neurol.

[59] Sevilis T, McDonald M, Avila A, Heath G, Gao L, O'Brien G, et al. Telestroke: maintaining quality acute stroke care during the COVID-19 pandemic. Telemed J E Health. 2022;28(4):481–5. 10.1089/tmj.2021.0149. 10.1089/tmj.2021.0149 PMC9058863 34265222

[60] Tavanaei R, Yazdani KO, Akhlaghpasand M, Zali A, Oraee-Yazdani S (2021). Changed pattern of hospital admission in stroke during COVID-19 pandemic period in Iran: a retrospective study. Neurol Sci.

[61] Srivastava PK, Zhang S, Xian Y, Xu H, Rutan C, Alger HM, et al. Treatment and outcomes of patients with ischemic stroke during COVID-19: an analysis from get with the guidelines-stroke. Stroke. 2021;52(10):3225-32. 10.1161/STROKEAHA.120.034414 PMC8478095 34192897

[62] Frisullo G, Brunetti V, Di Iorio R, Broccolini A, Caliandro P, Monforte M (2020). Effect of lockdown on the management of ischemic stroke: an Italian experience from a COVID hospital. Neurol Sci.

[63] Luo W, Li J, Li Z, Luo X, Chen M, Cai C (2021). Effects of the COVID-19 pandemic on reperfusion therapy for acute ischemic stroke patients in Huizhou City, China. Neurol Sci.

[64] Bhatia R, Sylaja PN, Srivastava MP, Komakula S, Iype T, Parthasarathy R (2021). Clinical profile and outcome of non-COVID strokes during pandemic and the pre pandemic period: COVID-stroke study group (CSSG) India. J Neurol Sci.

[65] Cummings C, Almallouhi E, Al Kasab S, Spiotta AM, Holmstedt CA (2020). Blacks are less likely to present with strokes during the COVID-19 pandemic: observations from the buckle of the stroke belt. Stroke..

[66] Rinkel LA, Prick JC, Slot RE, Sombroek NM, Burggraaff J, Groot AE (2021). Impact of the COVID-19 outbreak on acute stroke care. J Neurol.

[67] Ramos-Pachón A, García-Tornel Á, Millán M, Ribó M, Amaro S, Cardona P, et al. Bottlenecks in the acute stroke care system during the COVID-19 pandemic in catalonia. Cerebrovasc Dis. 2021;50(5):551–9. 10.1159/000516309. 10.1159/000516309 PMC8247826 34023822

[68] Tejada Meza H, Lambea Gil Á, Villar Yus C, Pérez Lázaro C, Navarro Pérez MP, Campello Morer I, et al. Three-month functional prognosis of patients hospitalised due to acute ischaemic stroke in Aragon: regional analysis of the impact of COVID-19. Neurologia (Engl Ed). 2021;36(7):531–6. 10.1016/j.nrleng.2021.02.001. 10.1016/j.nrleng.2021.02.001 PMC8441252 34099423

[69] Nagamine M, Chow DS, Chang PD, Boden-Albala B, Yu W and Soun JE. Impact of COVID-19 on acute stroke presentation at a comprehensive stroke center. Front. Neurol. 2020;11:850. 10.3389/fneur.2020.00850. 10.3389/fneur.2020.00850 PMC7456804 32922355

[70] Siegler JE, Zha AM, Czap AL, Ortega-Gutierrez S, Farooqui M, Liebeskind DS (2021). Influence of the COVID-19 pandemic on treatment times for acute ischemic stroke: the Society of Vascular and Interventional Neurology Multicenter collaboration. Stroke..

[71] Wang J, Chaudhry SA, Tahsili-Fahadan P, Altaweel LR, Bashir S, Bahiru Z (2020). The impact of COVID-19 on acute ischemic stroke admissions: analysis from a community-based tertiary care center. J Stroke Cerebrovasc Dis.

[72] Yang Y, Li Y, Du X (2021). Acute complex appendicitis during the COVID-19 epidemic: a single-institution retrospective analysis based on real-world data. Am J Emerg Med.

[73] Zhou Y, Cen LS (2020). Managing acute appendicitis during the COVID-19 pandemic in Jiaxing, China. World J Clin Cases.

[74] Tankel J, Keinan A, Blich O, Koussa M, Helou B, Shay S (2020). The decreasing incidence of acute appendicitis during COVID-19: a retrospective multi-Centre study. World J Surg.

[75] Orthopoulos G, Santone E, Izzo F, Tirabassi M, Pérez-Caraballo AM, Corriveau N (2021). Increasing incidence of complicated appendicitis during COVID-19 pandemic. Am J Surg.

[76] Kumaira Fonseca M, Trindade EN, Costa Filho OP, Nácul MP, Seabra AP (2020). Impact of COVID-19 outbreak on the emergency presentation of acute appendicitis. Am Surg.

[77] Turanli S, Kiziltan G (2021). Did the COVID-19 pandemic cause a delay in the diagnosis of acute appendicitis?. World J Surg.

[78] Wang AW, Prieto J, Ikeda DS, Lewis DOPR, Benzer DOEM, Van Gent DO (2021). Perforated appendicitis: an unintended consequence during the coronavirus-19 pandemic. Mil Med.

[79] Jäntti S, Ponkilainen V, Kuitunen I, Hevonkorpi TP, Paloneva J, Ukkonen M (2021). Trends in appendicectomy during the COVID-19 pandemic. Br J Surg.

[80] Lisi G, Campanelli M, Mastrangeli MR, Grande S, Viarengo MA, Garbarino GM, et al. Acute appendicitis in elderly during Covid-19 pandemic. Int J Colorectal Dis. 2021;36(10):2287–90. 10.1007/s00384-021-03959-x. 10.1007/s00384-021-03959-x PMC8159028 34046696

[81] Burgard M, Cherbanyk F, Nassiopoulos K, Malekzadeh S, Pugin F, Egger B (2021). An effect of the COVID-19 pandemic: significantly more complicated appendicitis due to delayed presentation of patients!. PLoS One.

[82] Antakia R, Xanthis A, Georgiades F, Hudson V, Ashcroft J, Rooney S (2021). Acute appendicitis management during the COVID-19 pandemic: a prospective cohort study from a large UK centre. Int J Surg.

[83] Finkelstein P, Picado O, Muddasani K, Wodnicki H, Mesko T, Unger S (2021). A retrospective analysis of the trends in acute appendicitis during the COVID-19 pandemic. J Laparoendosc Adv Surg Techn.

[84] Sartori A, Podda M, Botteri E, Passera R, Agresta F, Arezzo A; CRAC Study Collaboration Group. Appendectomy during the COVID-19 pandemic in Italy: a multicenter ambispective cohort study by the Italian Society of Endoscopic Surgery and new technologies (the CRAC study). Updates Surg. 2021;73(6):2205–13. 10.1007/s13304-021-01126-z. 10.1007/s13304-021-01126-z PMC8255092 34219197

[85] Baral S, Chhetri RK, Thapa N (2021). Comparison of acute appendicitis before and within lockdown period in COVID-19 era: a retrospective study from rural Nepal. PLoS One.

[86] Toale C, Westby D, O'Callaghan M, Nally D, Burke P, Peirce C (2020). Appendicitis and the COVID pandemic; new challenges in the management of a familiar foe. J Brit Surg.

[87] Dreifuss NH, Schlottmann F, Sadava EE, Rotholtz NA (2020). Acute appendicitis does not quarantine: surgical outcomes of laparoscopic appendectomy in COVID-19 times. J Brit Surg.

[88] Aharoni M, Barash Y, Zager Y, Anteby R, Khalilieh S, Amiel I, et al. Management of acute appendicitis during the COVID-19 pandemic: a single tertiary center experience. Isr Med Assoc J. 2021;23(5):269–73. 34024041

[89] Neufeld MY, Bauerle W, Eriksson E, Azar FK, Evans HL, Johnson M (2021). Where did the patients go? Changes in acute appendicitis presentation and severity of illness during the coronavirus disease 2019 pandemic: a retrospective cohort study. Surgery..

[90] Scheijmans JC, Borgstein AB, Puylaert CA, Bom WJ, Bachiri S, van Bodegraven EA (2021). Impact of the COVID-19 pandemic on incidence and severity of acute appendicitis: a comparison between 2019 and 2020. BMC Emerg Med.

[91] Somers K, Abd Elwahab S, Raza MZ, O'Grady S, DeMarchi J, Butt A, et al. Impact of the COVID-19 pandemic on management and outcomes in acute appendicitis: Should these new practices be the norm? Surgeon. 2021;19(5):e310–7. 10.1016/j.surge.2021.01.009. 10.1016/j.surge.2021.01.009 PMC7879062 33750630

[92] Barg M, Rotem R, Mor P, Rottenstreich M, Khatib F, Grisaru-Granovsky S (2021). Delayed presentation of ectopic pregnancy during the COVID-19 pandemic: a retrospective study of a collateral effect. Int J Gynecol Obstet.

[93] Dvash S, Cuckle H, Smorgick N, Vaknin Z, Padoa A, Maymon R (2021). Increase rate of ruptured tubal ectopic pregnancy during the COVID-19 pandemic. Eur J Obstet Gynecol Reprod Biol.

[94] Toma HV, Bank TC, Hoffman MK (2021). Care for women with ectopic pregnancies during the coronavirus disease 2019 (COVID-19) pandemic. Obstet Gynecol.

[95] Platts S, Ranawaka J, Oliver R, Patra-Das S, Kotabagi P, Neophytou C, et al. Impact of severe acute respiratory syndrome coronavirus 2 on ectopic pregnancy management in the United Kingdom: a multicentre observational study. BJOG. 2021;128(10):1625–34. 10.1111/1471-0528.16756. 10.1111/1471-0528.16756 PMC8209857 33998125

[96] Casadio P, Youssef A, Arena A, Gamal N, Pilu G, Seracchioli R. Increased rate of ruptured ectopic pregnancy in COVID-19 pandemic: analysis from the North of Italy. Ultrasound Obstet Gynecol. 2020;56(2):289. 10.1002/uog.22126. 10.1002/uog.22126 PMC7361714 32573042

[97] Anteby M, Van Mil L, Michaan N, Laskov I, Grisaru D (2021). Effects of the COVID-19 pandemic on timely care for extrauterine pregnancies: a retrospective analysis. Lancet Regional Health-Eur.

[98] Werner S, Katz A. Change in ectopic pregnancy presentations during the covid-19 pandemic. Int J Clin Pract. 2021;75(5):e13925. 10.1111/ijcp.13925. 10.1111/ijcp.13925 PMC7883085 33368867

[99] Dell’Utri C, Manzoni E, Cipriani S, Spizzico C, Dell’Acqua A, Barbara G (2020). Effects of SARS Cov-2 epidemic on the obstetrical and gynecological emergency service accesses. What happened and what shall we expect now?. Eur J Obstet Gynecol Reprod Biol.

[100] Tam CCF, Cheung KS, Lam S, Wong A, Yung A, Sze M (2020). Impact of coronavirus disease 2019 (COVID-19) outbreak on ST-segment–elevation myocardial infarction care in Hong Kong, China. Circul: Cardiovasc Qual Outcomes.

[101] Fileti L, Vecchio S, Moretti C, Reggi A, Aquilina M, Balducelli M (2020). Impact of the COVID-19 pandemic on coronary invasive procedures at two Italian high-volume referral centers. J Cardiovasc Med.

[102] Mesnier J, Cottin Y, Coste P, Ferrari E, Schiele F, Lemesle G (2020). Hospital admissions for acute myocardial infarction before and after lockdown according to regional prevalence of COVID-19 and patient profile in France: a registry study. Lancet Public Health.

[103] Choudhary R, Gautam D, Mathur R, Choudhary D (2020). Management of cardiovascular emergencies during the COVID-19 pandemic. Emerg Med J.

[104] Kwok CS, Gale CP, Kinnaird T, Curzen N, Ludman P, Kontopantelis E (2020). Impact of COVID-19 on percutaneous coronary intervention for ST-elevation myocardial infarction. Heart..

[105] Erol MK, Kayıkçıoğlu M, Kılıçkap M, Güler A, Yıldırım A, Kahraman F (2020). Treatment delays and in-hospital outcomes in acute myocardial infarction during the COVID-19 pandemic: a nationwide study. Anatolian J Cardiol.

[106] Toner L, Koshy AN, Hamilton GW, Clark D, Farouque O, Yudi MB (2020). Acute coronary syndromes undergoing percutaneous coronary intervention in the COVID-19 era: comparable case volumes but delayed symptom onset to hospital presentation. Euro Heart J-Qual Care Clin Outcomes.

[107] Li YH, Huang WC, Hwang JJ (2020). No reduction of ST-segment elevation myocardial infarction admission in Taiwan during coronavirus pandemic. Am J Cardiol.

[108] Claeys MJ, Argacha JF, Collart P, Carlier M, Van Caenegem O, Sinnaeve PR, et al. Impact of COVID-19-related public containment measures on the ST elevation myocardial infarction epidemic in Belgium: a nationwide, serial, cross-sectional study. Acta Cardiol. 2021;76(8):863–9. 10.1080/00015385.2020.1796035. 10.1080/00015385.2020.1796035 32727305

[109] Trabattoni D, Montorsi P, Merlino L (2020). Late STEMI and NSTEMI patients’ emergency calling in CoVID-19 outbreak. Can J Cardiol.

[110] Braiteh N, ur Rehman W, Alom M, Skovira V, Breiteh N, Rehman I (2020). Decrease in acute coronary syndrome presentations during the COVID-19 pandemic in upstate New York. Am Heart J.

[111] Scholz KH, Lengenfelder B, Thilo C, Jeron A, Stefanow S, Janssens U (2020). Impact of COVID-19 outbreak on regional STEMI care in Germany. Clin Res Cardiol.

[112] Hauguel-Moreau M, Pillière R, Prati G, Beaune S, Loeb T, Lannou S (2021). Impact of coronavirus disease 2019 outbreak on acute coronary syndrome admissions: four weeks to reverse the trend. J Thromb Thrombolysis.

[113] Romaguera R, Ribera A, Güell-Viaplana F, Tomás-Querol C, Muñoz-Camacho JF, Agudelo V (2020). Decrease in ST-segment elevation myocardial infarction admissions in Catalonia during the COVID-19 pandemic. Revista espanola de cardiologia (English ed).

[114] Xiang D, Xiang X, Zhang W, Yi S, Zhang J, Gu X (2020). Management and outcomes of patients with STEMI during the COVID-19 pandemic in China. J Am Coll Cardiol.

[115] Yasuda Y, Ishiguchi H, Ishikura M, Yoshida M, Imoto K, Sonoyama K, et al. Incidence and demographic trends for acute coronary syndrome in a non-epidemic area during the coronavirus disease pandemic in Japan - A 2-Institutional Observational Study. Circ Rep. 2021;3(2):95–9. 10.1253/circrep.CR-20-0141. 10.1253/circrep.CR-20-0141 PMC7939951 33693295

[116] Sutherland N, Dayawansa N, Filipopoulos B, Vasanthakumar S, Narayan O, Ponnuthurai FA, et al. Letter to the editor: acute coronary syndrome trends and COVID-19 waves (Response to the Letter of Čulić et al.). Heart Lung Circ. 2022;31(3):e34–5. 10.1016/j.hlc.2021.12.001. 10.1016/j.hlc.2021.12.001 PMC8754509 35033431

[117] Trabattoni D, Ravagnani PM, Merlino L, Montorsi P, Bartorelli AL (2021). The bimodal “rise and fall” ACS curve overlapping COVID-19 pandemic peaks. Am J Cardiovasc Dis.

[118] Calvão J, Amador AF, Costa CMD, Araújo PM, Pinho T, Freitas J, et al. The impact of the COVID-19 pandemic on acute coronary syndrome admissions to a tertiary care hospital in Portugal. Rev Port Cardiol (Engl Ed). 2021;41(2):147–52. 10.1016/j.repc.2021.01.007. 10.1016/j.repc.2021.01.007 PMC7980138 33892971

[119] Chan DZ, Stewart RA, Kerr AJ, Dicker B, Kyle CV, Adamson PD (2020). The impact of a national COVID-19 lockdown on acute coronary syndrome hospitalisations in New Zealand (ANZACS-QI 55). Lancet Regional Health-Western Pacific.

[120] Nan J, Meng S, Hu H, Jia R, Chen W, Li Q (2020). Comparison of clinical outcomes in patients with ST elevation myocardial infarction with percutaneous coronary intervention and the use of a telemedicine app before and after the COVID-19 pandemic at a Center in Beijing, China, from august 2019 to march 2020. Med Sci Monit: Int Med J Exper Clin Res.

[121] Tomasoni D, Adamo M, Italia L, Branca L, Chizzola G, Fiorina C (2020). Impact of COVID-2019 outbreak on prevalence, clinical presentation and outcomes of ST-elevation myocardial infarction. J Cardiovasc Med.

[122] Ojetti V, Covino M, Brigida M, Petruzziello C, Saviano A, Migneco A (2020). Non-COVID diseases during the pandemic: where have all other emergencies gone?. Medicina..

[123] Toniolo M, Negri F, Antonutti M, Masè M, Facchin D (2020). Unpredictable fall of severe emergent cardiovascular diseases hospital admissions during the COVID-19 pandemic: experience of a single large center in northern Italy. J Am Heart Assoc.

[124] Rattka M, Dreyhaupt J, Winsauer C, Stuhler L, Baumhardt M, Thiessen K (2021). Effect of the COVID-19 pandemic on mortality of patients with STEMI: a systematic review and meta-analysis. Heart..

[125] Anteby R, Zager Y, Barash Y, Nadler R, Cordoba M, Klang E (2020). The impact of the coronavirus disease 2019 outbreak on the attendance of patients with surgical complaints at a tertiary hospital emergency department. J Laparoendosc Adv Surg Techn.

[126] Madanelo M, Ferreira C, Nunes-Carneiro D, Pinto A, Rocha MA, Correia J, et al. The impact of the coronavirus disease 2019 pandemic on the utilisation of emergency urological services. BJU Int. 2020;126(2):256–8. 10.1111/bju.15109. 10.1111/bju.15109 PMC7272803 32406551

[127] Jeffery MM, D’Onofrio G, Paek H, Platts-Mills TF, Soares WE, Hoppe JA (2020). Trends in emergency department visits and hospital admissions in health care systems in 5 states in the first months of the COVID-19 pandemic in the US. JAMA Intern Med.

[128] Göksoy B, Akça MT, İnanç ÖF (2020). The impacts of the COVID-19 outbreak on emergency department visits of surgical patients. Turkish J Trauma Emerg Surg.

[129] Levitan EB, Howard VJ, Cushman M, Judd SE, Tison SE, Yuan Y (2021). Health care experiences during the COVID-19 pandemic by race and social determinants of health among adults age≥ 58 years in the REGARDS study. BMC Public Health.

[130] Andraska EA, Alabi O, Dorsey C, Erben Y, Velazquez G, Franco-Mesa C (2021). Health care disparities during the COVID-19 pandemic. Semin Vasc Surg.

[131] Kalani N, Hatami N, Haghbeen M, Yaqoob U, Raeyat Doost E (2021). COVID-19 health Care for Afghan Refugees as a minor ethnicity in Iran: clinical differences and racial equality in health. Acta Med Iran.

[132] Okazaki T, Kuroda Y (2018). Aneurysmal subarachnoid hemorrhage: intensive care for improving neurological outcome. J Intensive Care.

[133] D’Souza S (2015). Aneurysmal subarachnoid hemorrhage. J Neurosurg Anesthesiol.

[134] Peeri NC, Shrestha N, Rahman MS, Zaki R, Tan Z, Bibi S (2020). The SARS, MERS and novel coronavirus (COVID-19) epidemics, the newest and biggest global health threats: what lessons have we learned?. Int J Epidemiol.

[135] Lazzeroni P, Bernardi L, Pecora F, Motta M, Bianchi L, Ruozi MB, et al. Diabetic ketoacidosis at type 1 diabetes onset: indirect impact of COVID-19 pandemic. Acta Bio Medica: Atenei Parmensis. 2020;91(4). 10.23750/abm.v91i4.10943 PMC7927550 33525255

[136] Gayoso M, Lim WY, Mulekar MS, Kaulfers AM (2021). Effect of Covid-19 quarantine on diabetes Care in Children. Clin Diab Endocrinol.

[137] Burkart S, Parker H, Weaver RG, Beets MW, Jones A, Adams EL, et al. Impact of the COVID-19 pandemic on elementary schoolers' physical activity, sleep, screen time and diet: a quasi-experimental interrupted time series study. Pediatr Obes. 2021:e12846. 10.1111/ijpo.12846 PMC8420216 34409754

[138] Varadarajan P, Suresh S (2015). Delayed diagnosis of diabetic ketoacidosis in children—a cause for concern. Int J Diabetes Dev Countries.

[139] Cefalu WT, Smith SR, Blonde L, Fonseca V (2006). The hurricane Katrina aftermath and its impact on diabetes care: observations from “ground zero”: lessons in disaster preparedness of people with diabetes. Diabetes Care.

[140] Carameli KA, Eisenman DP, Blevins J, d'Angona B, Glik DC (2013). Planning for chronic disease medications in disaster: perspectives from patients, physicians, pharmacists, and insurers. Disast Med Public Health Preparedness.

[141] Place R, Lee J, Howell J (2020). Rate of pediatric appendiceal perforation at a children’s hospital during the COVID-19 pandemic compared with the previous year. JAMA Netw Open.

