# Supplementary material for: Global burden of the COVID-19 associated patient-related delay in emergency healthcare: a panel of systematic review and meta-analyses
Source: Global Health. 2022 Jun 8;18:58. doi: 10.1186/s12992-022-00836-2 (PMC9175527; doi:10.1186/s12992-022-00836-2)

**Supplementary tables and figures**

Sup table 1. Search strategy

|  | Search strategy | screened items | items selected for abstract review |
| --- | --- | --- | --- |
| meningitis | (("Meningitis"[MeSH Major Topic] AND "COVID-19"[Title]) OR "SARS-COV2"[Title] OR "SARS-COV-2"[Title] OR "Pandemic"[Title] OR "Quarantine"[Title]) AND "Meningitis"[Title] | 23 | 9 |
| Stroke | (("stroke"[MeSH Major Topic] AND "COVID-19"[Title]) OR "SARS-COV2"[Title] OR "SARS-COV-2"[Title] OR "Pandemic"[Title] OR "Quarantine"[Title]) AND "Stroke"[Title] | 205 | 95 |
| seizures | ((((("Seizures"[MeSH Major Topic] AND "COVID-19"[Title]) OR "SARS-COV2"[Title] OR "SARS-COV-2"[Title] OR "Pandemic"[Title] OR "Quarantine"[Title]) AND "Non-convulsive ‎status epilepticus"[Title] AND (alladult[Filter])) NOT (Case Report[Title]) AND (alladult[Filter]) ) NOT (Case Series[Title]) AND (alladult[Filter])) AND (Non-convulsive ‎status epilepticus) | 6 | 0 |
|  | ((((("Seizures"[MeSH Major Topic] AND "COVID-19"[Title]) OR "SARS-COV2"[Title] OR "SARS-COV-2"[Title] OR "Pandemic"[Title] OR "Quarantine"[Title]) AND "Seizures"[Title] AND (Non-convulsive ‎status epilepticus) | 2 | 0 |
| aneurysm | (("aneurysm"[MeSH Major Topic] AND "COVID-19"[Title]) OR "SARS-COV2"[Title] OR "SARS-COV-2"[Title] OR "Pandemic"[Title] OR "Quarantine"[Title]) AND "Aneurysm"[Title] | 25 | 15 |
| Cardiac Tamponade | ((("Cardiac Tamponade"[MeSH Major Topic] AND "COVID-19"[Title]) OR "SARS-COV2"[Title] OR "SARS-COV-2"[Title] OR "Pandemic"[Title] OR "Quarantine"[Title]) AND "Cardiac Tamponade"[Title]) AND (Tamponade[Title]) NOT (Case Report[Title]) AND (alladult[Filter]) ) NOT (Case Series[Title]) AND (alladult[Filter])) | 9 | 0 |
| gastrointestinal hemorrhage | ((((((gastrointestinal hemorrhage[MeSH Major Topic]) AND (COVID-19[Title])) OR (SARS-COV2[Title])) OR (SARS-COV-2[Title])) OR (Pandemic[Title])) OR (Quarantine[Title])) AND (gastrointestinal hemorrhage) | 33 | 5 |
| Diabetic Ketoacidosis | (("Diabetes Mellitus"[MeSH Major Topic] AND ("COVID-19"[Title] OR "SARS-COV2"[Title] OR "SARS-COV-2"[Title] OR "Pandemic"[Title] OR "Quarantine"[Title]) AND "Diabetic Ketoacidosis"[Title] | 24 | 7 |
|  | (("Diabetes Mellitus"[MeSH Major Topic] AND ("COVID-19"[Title] OR "SARS-COV2"[Title] OR "SARS-COV-2"[Title] OR "Pandemic"[Title] OR "Quarantine"[Title]) AND ("Diabetic Ketoacidosis"[Title] OR "type 1 diabetes"[Title]) | 74 | 45 |
| Hypoglycemia | (("Diabetes Mellitus"[MeSH Major Topic] AND ("COVID-19"[Title] OR "SARS-COV2"[Title] OR "SARS-COV-2"[Title] OR "Pandemic"[Title] OR "Quarantine"[Title]) AND ("Hypoglycemia"[Title]) AND (alladult[Filter]) | 4 | 2 |
| Addison's and adrenocortical insufficiency | ("COVID-19"[Title] OR "SARS-COV2"[Title] OR "SARS-COV-2"[Title] OR "Pandemic"[Title] OR "Quarantine"[Title]) AND ("Addison's?"[Title]) | 0 | 0 |
|  | ("COVID-19"[Title] OR "SARS-COV2"[Title] OR "SARS-COV-2"[Title] OR "Pandemic"[Title] OR "Quarantine"[Title]) AND ("adrenocortical ‎insufficiency"[Title]) | 0 | 0 |
|  | (("Adrenal Cortex Hormones"[MeSH Major Topic] AND ("COVID-19"[Title] OR "SARS-COV2"[Title] OR "SARS-COV-2"[Title] OR "Pandemic"[Title] OR "Quarantine"[Title]) AND ("adrenocortical ‎insufficiency"[Title]) AND (alladult[Filter]) AND (alladult[Filter]) | 0 | 0 |
| Phaeochromocytoma | ("COVID-19"[Title] OR "SARS-COV2"[Title] OR "SARS-COV-2"[Title] OR "Pandemic"[Title] OR "Quarantine"[Title]) AND ("Phaeochromocytoma"[Title]) AND (alladult[Filter]) | 0 | 0 |
| Hypercalcaemia | ("COVID-19"[Title] OR "SARS-COV2"[Title] OR "SARS-COV-2"[Title] OR "Pandemic"[Title] OR "Quarantine"[Title]) AND ("Hypercalcaemia"[Title]) | 0 | 0 |
| Thyroid Crisis | (("Thyroid Crisis"[MeSH Major Topic] AND ("COVID-19"[Title] OR "SARS-COV2"[Title] OR "SARS-COV-2"[Title] OR "Pandemic"[Title] OR "Quarantine"[Title]) AND ("Thyroid Crisis"[Title]) | 0 | 0 |
|  | (("Thyroid Crisis"[MeSH Major Topic] AND ("COVID-19"[Title] OR "SARS-COV2"[Title] OR "SARS-COV-2"[Title] OR "Pandemic"[Title] OR "Quarantine"[Title]) AND ("Thyroid"[Title]) | 0 | 0 |
| Scleroderma | (("Connective Tissue Diseases"[MeSH Major Topic] AND ("COVID-19"[Title] OR "SARS-COV2"[Title] OR "SARS-COV-2"[Title] OR "Pandemic"[Title] OR "Quarantine"[Title]) AND ("Scleroderma ‎"[Title]) | 4 | 0 |
| Vasculitis | (("Vasculitis"[MeSH Major Topic] AND ("COVID-19"[Title] OR "SARS-COV2"[Title] OR "SARS-COV-2"[Title] OR "Pandemic"[Title] OR "Quarantine"[Title]) AND ("Vasculitis?"[Title]) | 41 | 3 |
| Myxedema | (("Connective Tissue Diseases"[MeSH Major Topic] AND ("COVID-19"[Title] OR "SARS-COV2"[Title] OR "SARS-COV-2"[Title] OR "Pandemic"[Title] OR "Quarantine"[Title]) AND ("Myxedema"[Title]) | 0 | 0 |
| Nephrotic Syndrome | (("Kidney Diseases"[MeSH Major Topic] AND ("COVID-19"[Title] OR "SARS-COV2"[Title] OR "SARS-COV-2"[Title] OR "Pandemic"[Title] OR "Quarantine"[Title]) AND ("Nephrotic Syndrome"[Title]) | 6 | 0 |
|  | (("Kidney Diseases"[MeSH Major Topic] AND ("COVID-19"[Title] OR "SARS-COV2"[Title] OR "SARS-COV-2"[Title] OR "Pandemic"[Title] OR "Quarantine"[Title]) AND ("Nephrotic Syndrome"[Title]) | 1 | 0 |
| Appendicitis | (("Abdominal Pain"[MeSH Major Topic] AND ("COVID-19"[Title] OR "SARS-COV2"[Title] OR "SARS-COV-2"[Title] OR "Pandemic"[Title] OR "Quarantine"[Title]) AND ("Acute"[Title]) | 11 | 1 |
|  | ("COVID-19"[Title] OR "SARS-COV2"[Title] OR "SARS-COV-2"[Title] OR "Pandemic"[Title] OR "Quarantine"[Title]) AND ("Acute abdomen"[Title] OR "Abdominal pain"[Title] OR "Appendicitis"[Title] AND ((alladult[Filter]) AND (2019:2022[pdat])) AND (alladult[Filter]) | 501 | 31 |
| Hernia | ("COVID-19"[Title] OR "SARS-COV2"[Title] OR "SARS-COV-2"[Title] OR "Pandemic"[Title] OR "Quarantine"[Title]) AND ("Acute abdomen"[Title] OR "Abdominal pain"[Title] OR "Appendicitis"[Title] AND ((alladult[Filter]) AND (2019:2022[pdat])) AND (alladult[Filter]) | 501 | 31 |
| ACS | ((("Heart Diseases"[MeSH Major Topic] AND "COVID-19"[Title]) OR "SARS-COV2"[Title] OR "SARS-COV-2"[Title] OR "Pandemic"[Title] OR "Quarantine"[Title]) AND "Myocardial Infarction"[Title]) AND (ST Elevation Myocardial Infarction[Title]) NOT (Case Report[Title]) AND (alladult[Filter]) ) NOT (Case Series[Title]) AND (alladult[Filter])) | 23 | 8 |
|  | ("COVID-19"[Title] OR "SARS-COV2"[Title] OR "SARS-COV-2"[Title] OR "Pandemic"[Title] OR "Quarantine"[Title]) AND "Acute Coronary Syndrome"[Title]) | 71 | 38 |
| Obstruction | ("COVID-19"[Title] OR "SARS-COV2"[Title] OR "SARS-COV-2"[Title] OR "Pandemic"[Title] OR "Quarantine"[Title]) AND ("Intestinal ‎"[Title] OR "Intestinal Obstruction"[Title] OR "Obstruction"[Title] | 1482 | 1 |
| Esophageal varices | (("Esophageal Diseases"[MeSH Major Topic] AND ("COVID-19"[Title] OR "SARS-COV2"[Title] OR "SARS-COV-2"[Title] OR "Pandemic"[Title] OR "Quarantine"[Title]) AND ("bleeding"[Title] OR "varices"[Title] OR "esophageal varices"[Title] AND (alladult[Filter]) | 205 | 2 |
| Ectopic pregnancy | ("COVID-19"[Title] OR "SARS-COV2"[Title] OR "SARS-COV-2"[Title] OR "Pandemic"[Title] OR "Quarantine"[Title]) AND ("Ectopic Pregnancy?"[Title] OR "Ectopic"[Title] OR "Pregnancy Complications"[Title] | 603 | 5 |
| ovarian torsion | ("COVID-19"[Title] OR "SARS-COV2"[Title] OR "SARS-COV-2"[Title] OR "Pandemic"[Title] OR "Quarantine"[Title]) AND ("Adnexal Torsion"[Title] OR "Ovarian Torsion"[Title] OR "Torsion"[Title] | 20 | 0 |
|  | ((("Ovarian Diseases"[MeSH Major Topic] AND ("COVID-19"[Title] OR "SARS-COV2"[Title] OR "SARS-COV-2"[Title] OR "Pandemic"[Title] OR "Quarantine"[Title]) AND ("Adnexal Torsions"[Title] OR "Ovarian "[Title] OR "Ovarian Torsions"[Title] OR "Torsion"[Title] AND ((alladult[Filter]) AND (2019:2022[pdat]))) AND Covid-19 | 10 | 0 |
| abortion | ((("Pregnancy Complications"[MeSH Major Topic] AND ("COVID-19"[Title] OR "SARS-COV2"[Title] OR "SARS-COV-2"[Title] OR "Pandemic"[Title] OR "Quarantine"[Title]) AND ("Miscarriage"[Title] OR "Spontaneous"[Title] OR "Abortion"[Title] OR "Pregnancy Complications"[Title] AND Covid-19 NOT "pneumothorax" NOT "pneumomediastinum" | 261 | 70 |
| PROM | ("COVID-19"[Title] OR "SARS-COV2"[Title] OR "SARS-COV-2"[Title] OR "Pandemic"[Title] OR "Quarantine"[Title]) AND ("PROM "[Title] OR "Premature Rupture of Fetal Membranes"[Title] OR "Premature Rupture"[Title] OR "Fetal Membranes"[Title] AND ((alladult[Filter]) | 79 | 0 |
| Urinary Retention | ("COVID-19"[Title] OR "SARS-COV2"[Title] OR "SARS-COV-2"[Title] OR "Pandemic"[Title] OR "Quarantine"[Title]) AND ("PROM "[Title] OR "Premature Rupture of Fetal Membranes"[Title] OR "Premature Rupture"[Title] OR "Fetal Membranes"[Title] AND ((alladult[Filter]) | 245 | 25 |
| Fournier Gangrene | Bacterial Infections[MeSH Major Topic] AND ("COVID-19"[Title] OR "SARS-COV2"[Title] OR "SARS-COV-2"[Title] OR "Pandemic"[Title] OR "Quarantine"[Title]) AND (("Gangrene"[Title] OR " Fournier Gangrene"[Title] OR "Fournier "[Title]) | 3 | 0 |
| Hematuria | Urologic Diseases[MeSH Major Topic] AND ("COVID-19"[Title] OR "SARS-COV2"[Title] OR "SARS-COV-2"[Title] OR "Pandemic"[Title] OR "Quarantine"[Title]) AND (("Acute"[Title] OR " Urinary "[Title] OR "Hematuria"[Title] OR "Severe Hematuria"[Title]) NOT " COVID-19 Patients" NOT "Patients with COVID-19" NOT "COVID-19 associated" NOT "with COVID-19" | 98 | 27 |

Supplementary Fig 1. Forrest plot of CVA symptoms onset to ED door time


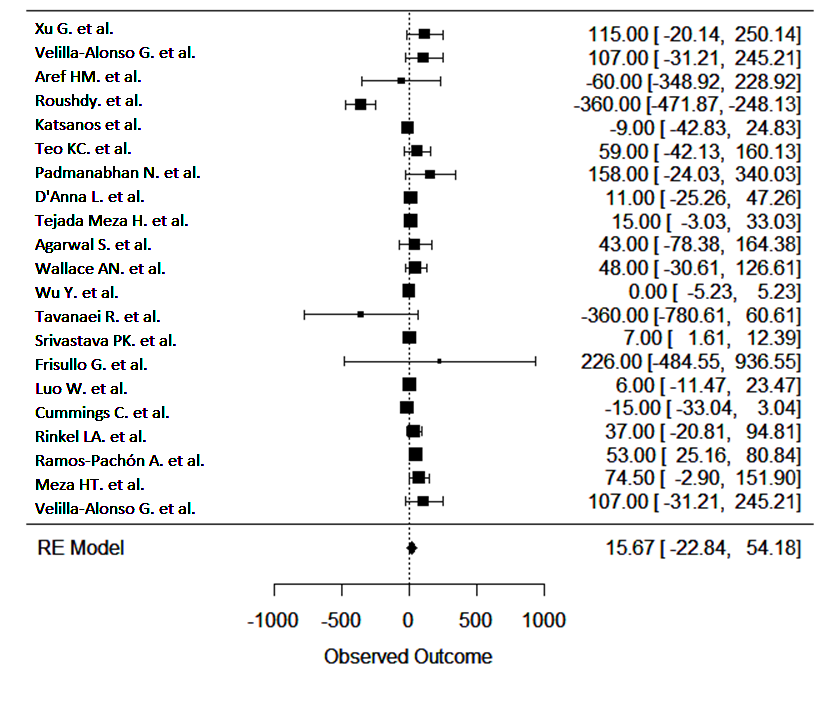


Supplementary Fig 2. Funnel plot of CVA symptoms onset to ED door time


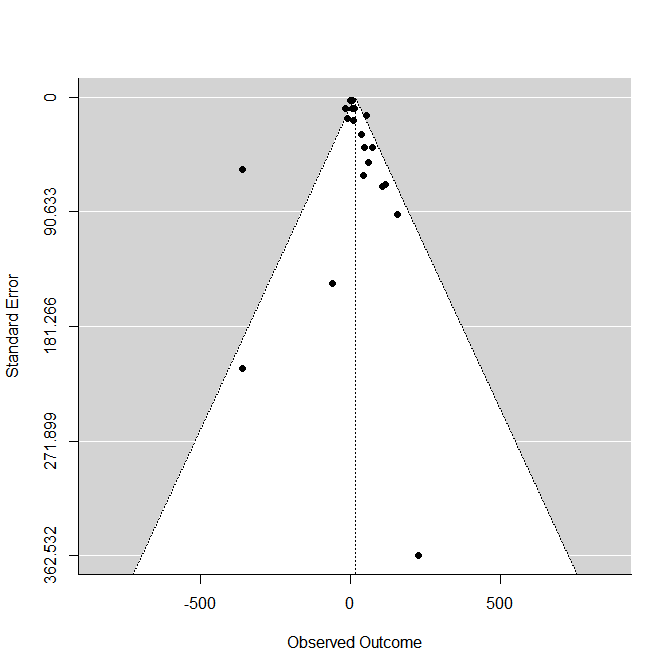


Supplementary Fig 3. Forrest plot of rt-PA administration proportion
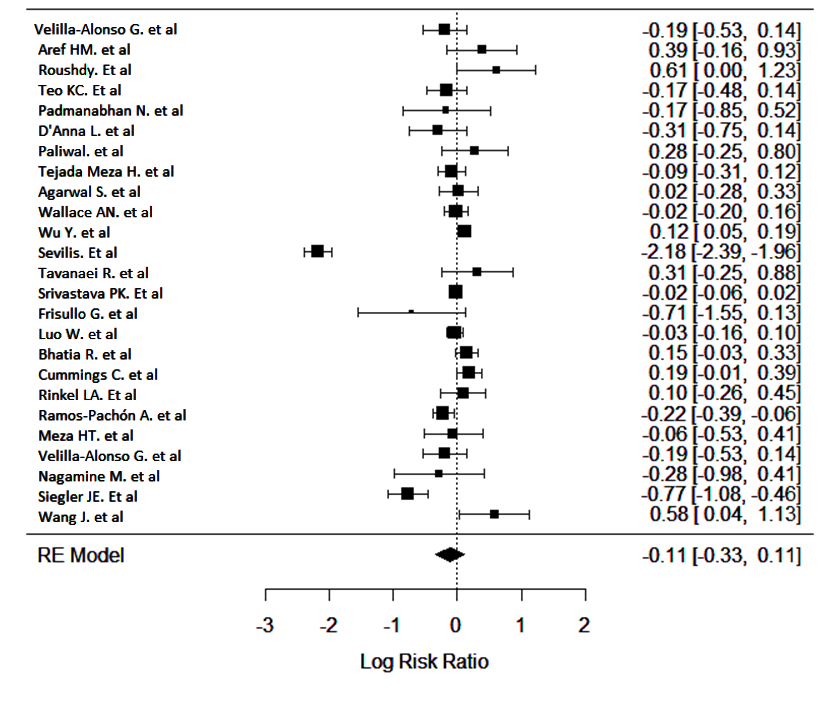


Supplementary Fig 4. Funnel plot of rt-PA administration proportion
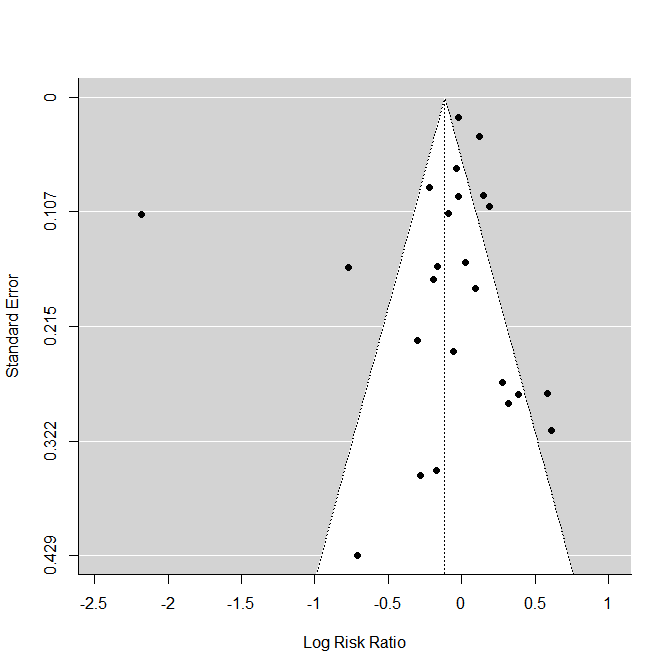


Supplementary Fig 5. Forest and Funnel plot of SAH Vasospasm


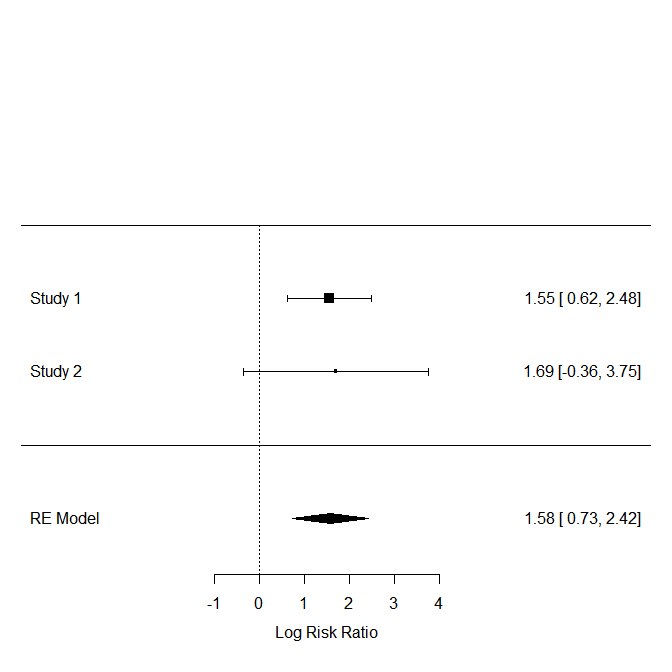

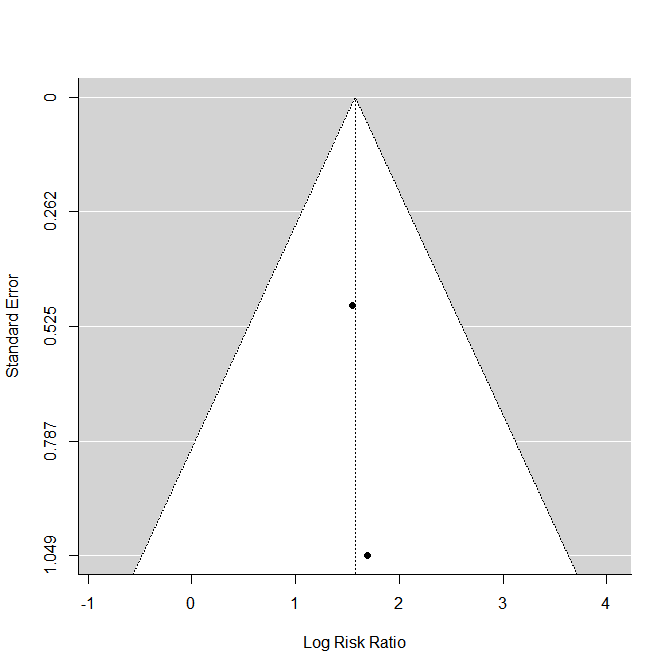


(study 1, Fiorindi et al.; study 2, Aboukaïs et al.)

Supplementary Fig 6. Forest and Funnel plot of Fisher grade>2


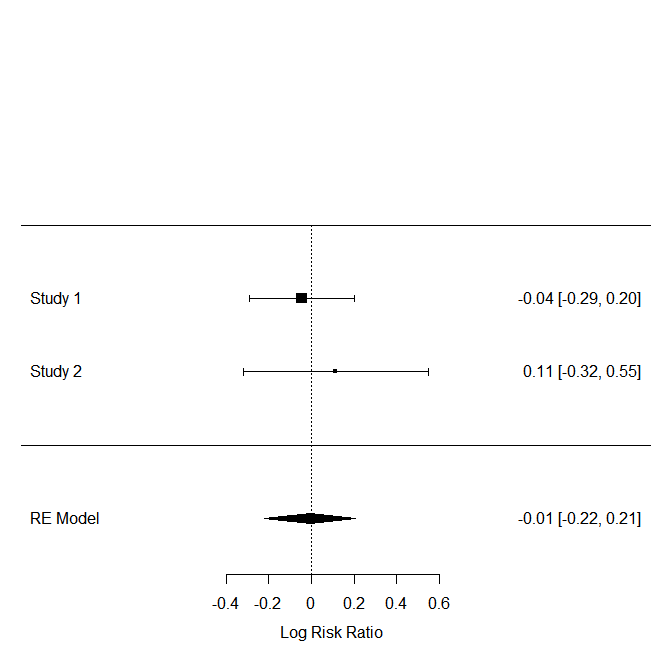

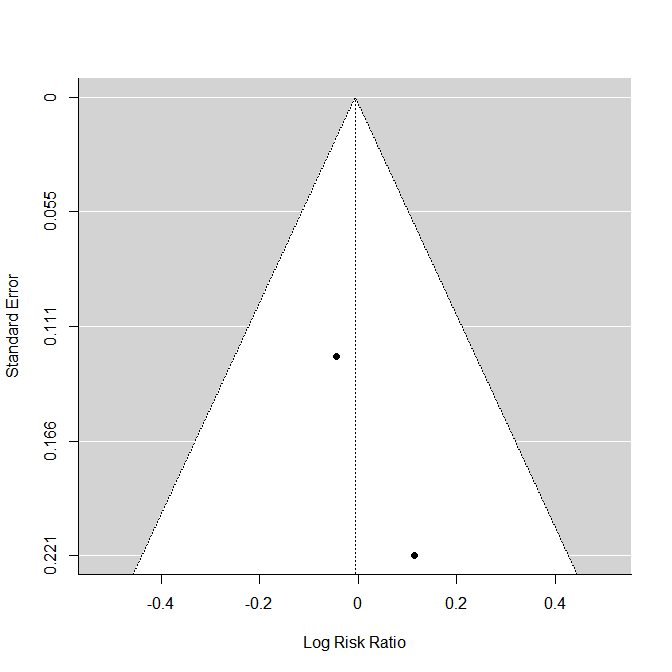


(study 1, Fiorindi et al.; study 2, Aboukaïs et al.)

Supplementary Fig 7. Forest and Funnel plot of WFNS > 3


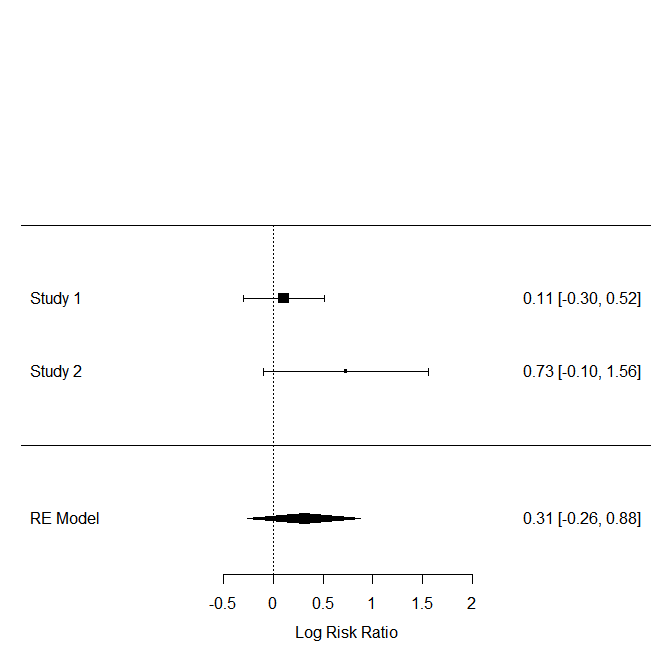

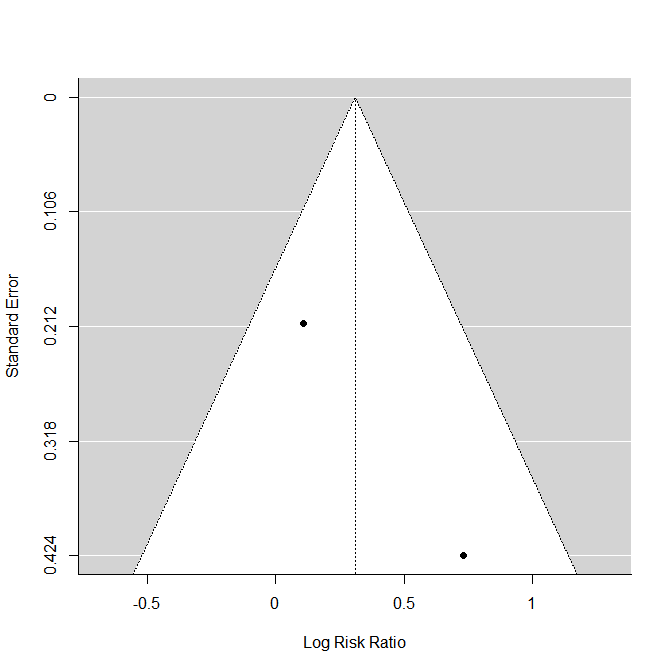


(study 1, Fiorindi et al.; study 2, Aboukaïs et al.)

Supplementary Fig 8. Forrest plot of Orchiectomy rate in testicular torsion
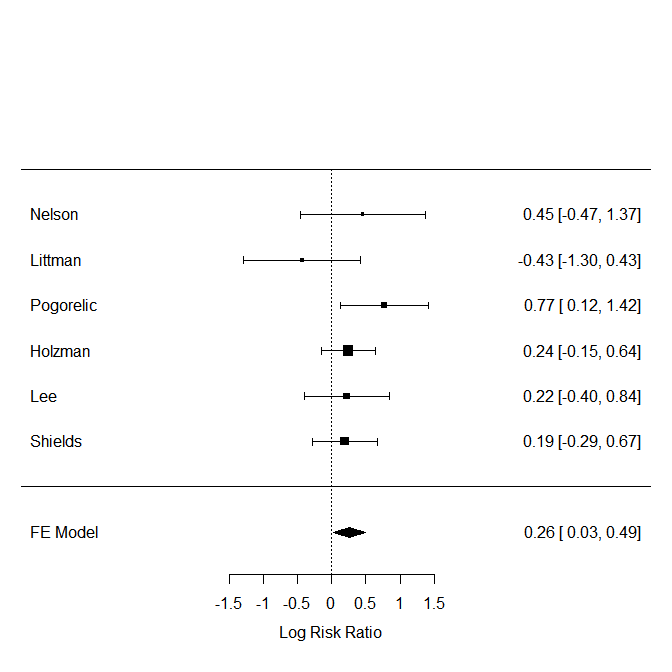


Supplementary Fig 9. Funnel plot of Orchiectomy rate in testicular torsion


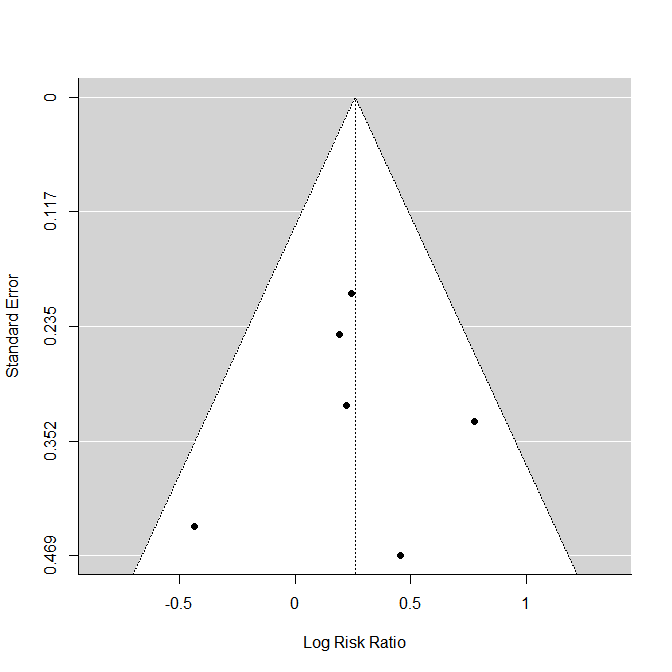


Supplementary Fig 10. Forrest plot of DKA presentation among newly diagnosed T1DM patients


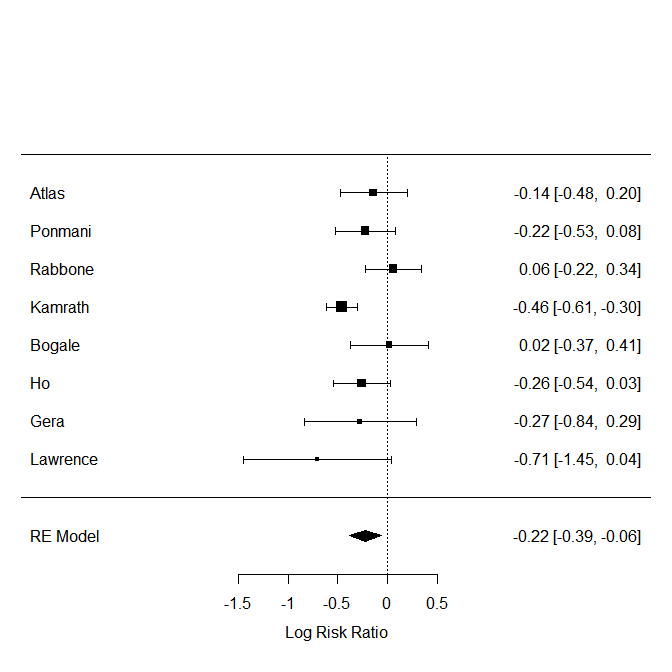


Supplementary Fig 11. Funnel plot of DKA presentation among newly diagnosed T1DM patients


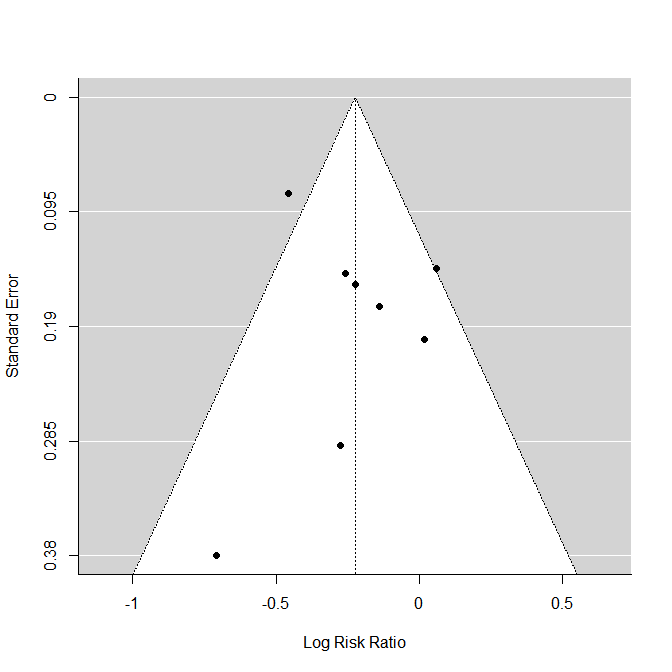


Supplementary Fig 12. Forrest plot of Perforated ectopic pregnancy


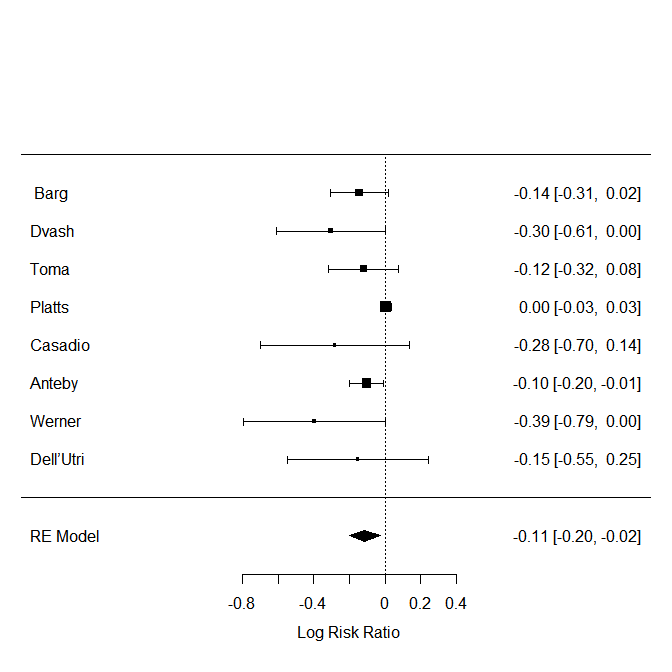


Supplementary Fig 13. Funnel plot of Perforated ectopic pregnancy


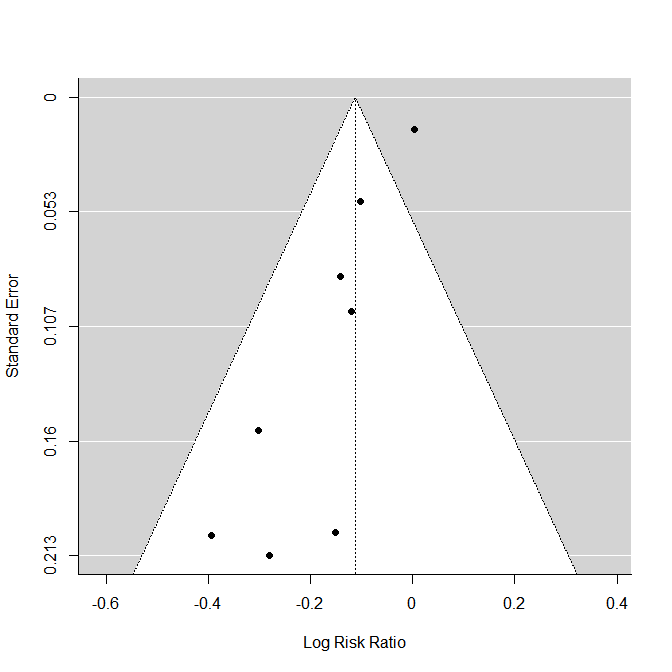


Supplementary Fig 14. trim-filled Forrest plot of Perforated ectopic pregnancy


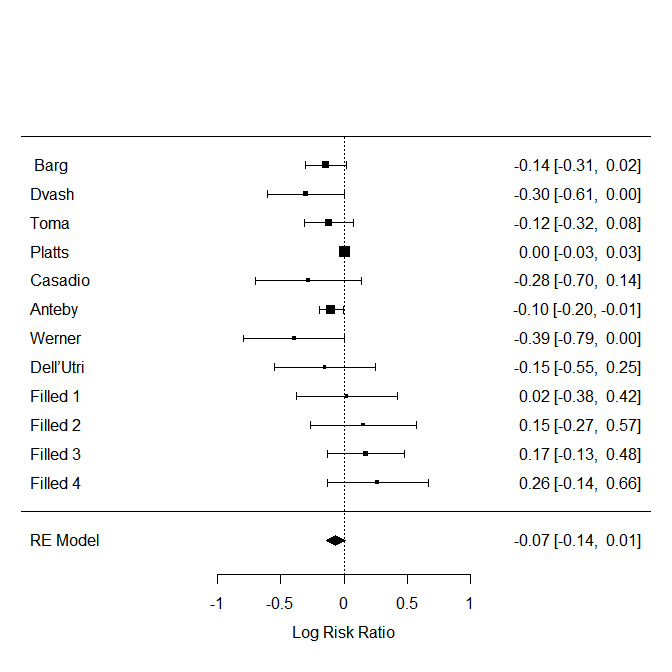


Supplementary Fig 15. trim-filled Funnel plot of Perforated ectopic pregnancy


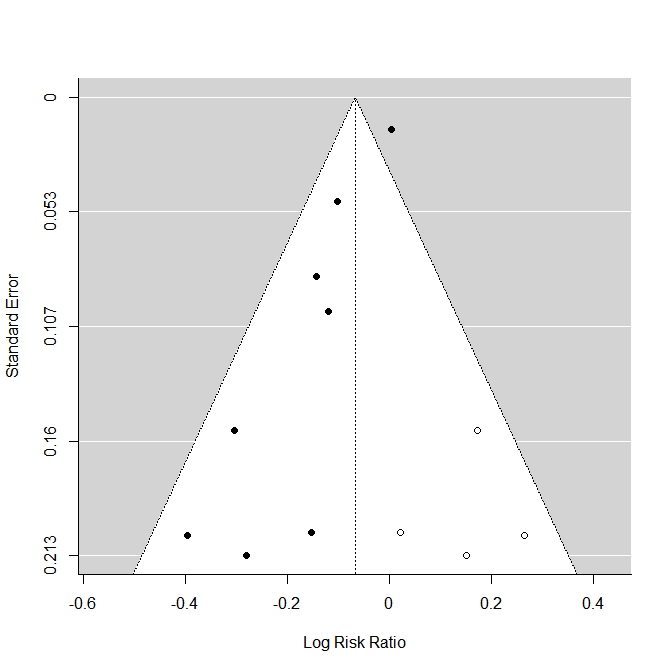


Supplementary Fig 16. Forest plot of perforated appendicitis proportion


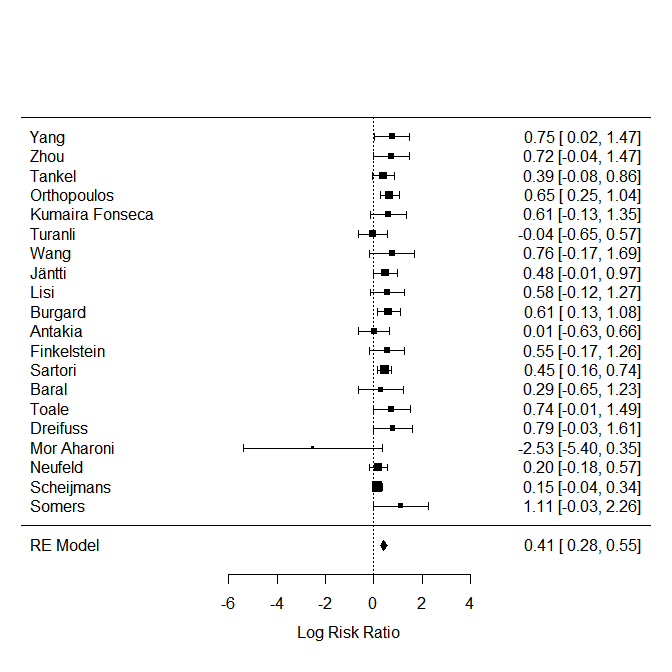


Supplementary Fig 17. Funnel plot of perforated appendicitis proportion


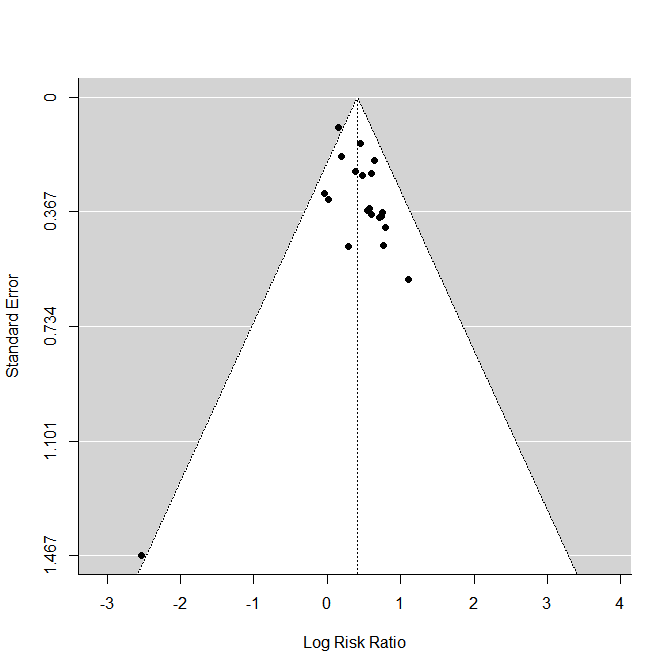


Supplementary Fig 18. Forest plot of delayed appendicitis presentation


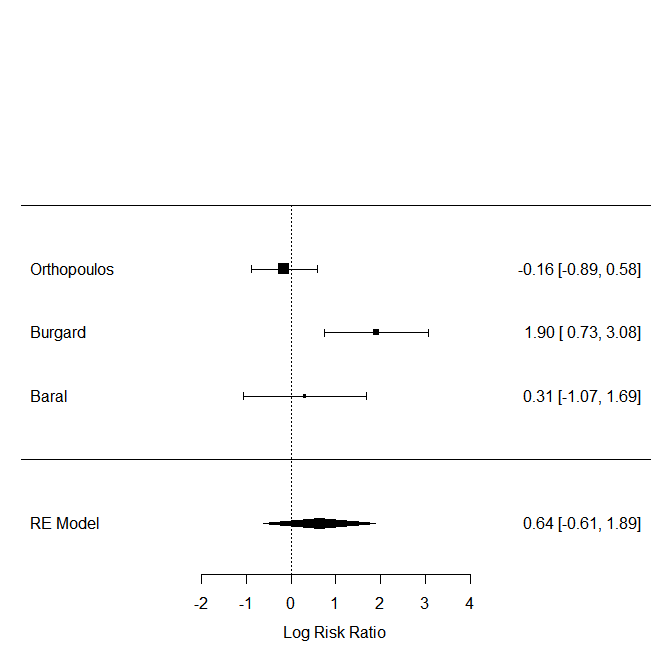


Supplementary Fig 19. Funnel plot of delayed appendicitis presentation


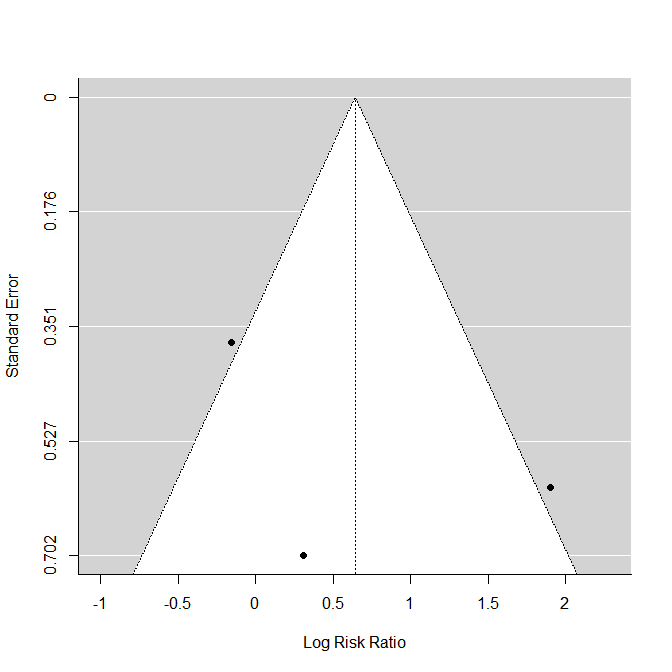

Supplement: Supplementary file 1 — Additional file 1: Sup table 1. Search strategy. Supplementary Fig. 1. Forrest plot of CVA symptoms onset to ED door time. Supplementary Fig. 2. Funnel plot of CVA symptoms onset to ED door time. Supplementary Fig. 3. Forrest plot of rt-PA administration proportion. Supplementary Fig. 4. Funnel plot of rt-PA administration proportion . Supplementary Fig. 5. Forest and Funnel plot of SAH Vasospasm (study 1, Fiorindi et al.; study 2, Aboukaïs et al.). Supplementary Fig. 6. Forest and Funnel plot of Fisher grade > 2 (study 1, Fiorindi et al.; study 2, Aboukaïs et al.). Supplementary Fig. 7. Forest and Funnel plot of WFNS > 3 (study 1, Fiorindi et al.; study 2, Aboukaïs et al.). Supplementary Fig. 8. Forrest plot of Orchiectomy rate in testicular torsion. Supplementary Fig. 9. Funnel plot of Orchiectomy rate in testicular torsion. Supplementary Fig. 10. Forrest plot of DKA presentation among newly diagnosed T1DM patients. Supplementary Fig. 11. Funnel plot of DKA presentation among newly diagnosed T1DM patients. Supplementary Fig. 12. Forrest plot of Perforated ectopic pregnancy. Supplementary Fig. 13. Funnel plot of Perforated ectopic pregnancy. Supplementary Fig. 14. trim-filled Forrest plot of Perforated ectopic pregnancy. Supplementary Fig. 15. trim-filled Funnel plot of Perforated ectopic pregnancy. Supplementary Fig. 16. Forest plot of perforated appendicitis proportion. Supplementary Fig. 17. Funnel plot of perforated appendicitis proportion. Supplementary Fig. 18. Forest plot of delayed appendicitis presentation. Supplementary Fig. 19. Funnel plot of delayed appendicitis presentation. [file 12992_2022_836_MOESM1_ESM.docx]
